# Degradable Poly(3-hydroxybutyrate)—The Basis of Slow-Release Fungicide Formulations for Suppressing Potato Pathogens

**DOI:** 10.3390/polym14173669

**Published:** 2022-09-03

**Authors:** Tatiana G. Volova, Evgeniy G. Kiselev, Sergey V. Baranovskiy, Natalia O. Zhila, Svetlana V. Prudnikova, Ekaterina I. Shishatskaya, Andrey P. Kuzmin, Ivan V. Nemtsev, Aleksander D. Vasiliev, Sabu Thomas

**Affiliations:** 1 Basic Department of Biotechnology, School of Fundamental Biology and Biotechnology, Siberian Federal University, 79 Svobodnyi Av., 660041 Krasnoyarsk, Russia; 2 Institute of Biophysics SB RAS, Federal Research Center “Krasnoyarsk Science Center SB RAS”, 50/50 Akademgorodok, 660036 Krasnoyarsk, Russia; 3 Department of Medical Biology, School of Fundamental Biology and Biotechnology, Siberian Federal University, 79 Svobodnyi Av., 660041 Krasnoyarsk, Russia; 4 Basic Department of Chemistry and Technology of Natural Energy Sources and Carbon Materials, School of Petroleum and Gas Engineering, Siberian Federal University, 82 Svobodny Pr., 660041 Krasnoyarsk, Russia; 5 L.V. Kirensky Institute of Physics SB RAS, Federal Research Center “Krasnoyarsk Science Center SB RAS”, 50/38 Akademgorodok, 660036 Krasnoyarsk, Russia; 6 Federal Research Center, “Krasnoyarsk Science Center of the Siberian Branch of the Russian Academy of Sciences”, 50 Akademgorodok, 660036 Krasnoyarsk, Russia; 7 Basic Department of Solid State Physics and Nanotechnology, School of Engineering Physics and Radio Electronics, Siberian Federal University, 26 Kirensky St., 660074 Krasnoyarsk, Russia; 8 International and Interuniversity Centre for Nano Science and Nano Technology, Mahatma Gandhi University, Kottayam 686560, India

**Keywords:** fungicides, degradable P(3HB), slow-release formulations, physico-chemical properties, degradation in soil, suppression of plant pathogens

## Abstract

Three-component slow-release fungicide formulations with different modes of action of the active ingredients for suppressing potato pathogens were constructed for the first time. The difenoconazole, mefenoxam, prothioconazole, and azoxystrobin fungicides were embedded in the degradable polymer P(3HB)/birch wood flour blend and examined using SEM, IR spectroscopy, X-ray analysis, DTA, and DSC. Results showed that no chemical bonds were established between the components and that they were physical mixtures that had a lower degree of crystallinity compared to the initial P(3HB), which suggested different crystallization kinetics in the mixtures. The degradation behavior of the experimental formulations was investigated in laboratory micro-ecosystems with pre-characterized field soil. The slow-release fungicide formulations were prolonged-action forms with a half-life of at least 50–60 d, enabling gradual and sustained delivery of the active ingredients to plants. All slow-release fungicide formulations had a strong inhibitory effect on the most common and harmful potato pathogens (*Phytophthora* *infestans*, *Alternaria* *longipes*, *Rhizoctonia* *solani*, and *Fusarium* *solani*).

## 1. Introduction

The traditional extensive use of chemically synthesized products, which are derived from nonrenewable natural resources, has led to the accumulation of non-recyclable waste, contravening environmental protection measures and posing a global problem [1,2,3]. One of the approaches to alleviating human impact on ecosystems is to develop new environmentally friendly compounds and materials and use them efficiently [4].

Increasing importance has been attached recently to industrial ecology and green chemistry, which aim to create new environmentally friendly materials and life support products from renewable sources [5]. These approaches were developed in order to resolve global environmental issues caused by products of the technosphere such as xenobiotics and synthetic non-degradable plastics accumulated in the biosphere. Thus, research aimed at construction and various applications of hybrid materials consisting of synthetic polymers and widely available natural materials (various plant wastes, peat, clay, etc.) has received increased attention [4,6,7]. Degradable polymers synthesized by living systems, the so-called biopolymers, are very promising materials for such composites [8,9].

Polyhydroxyalkanoates (PHAs)—polymers of microbial origin—hold a special place among natural degradable materials. PHAs are biodegradable thermoplastic polymers varying in their chemical composition and physicochemical properties. These polymers are processable from different phase states (solution, emulsion, powder, melt) by available techniques [10,11,12]. In addition, they can be used to produce composites with different fillers and materials [13,14,15,16,17,18]. Therefore, PHAs are promising materials for various applications—from municipal engineering and agriculture to medicine and pharmacology [19,20,21], with good potential as contributors to “The Circular Economy” [22]. PHAs are prospective materials for reconstructive medicine, cellular and tissue engineering, and controlled delivery of drugs and bioactive substances. These are high-value-added products of small-scale industries [12,23].

The high cost of PHAs limits their use in large-scale production of degradable packaging, household and construction goods, film and pots for greenhouses, and systems for delivery of fertilizers and pesticides. To make PHAs less expensive and more widely available, different approaches are tried to improve PHA production processes, including the use of cheaper substrates [24]. Another way to reduce the cost of PHAs is to blend these polymers with inexpensive natural materials. Constructing PHA-based composites is a way to not only reduce their cost but also alter and improve their basic properties. There are numerous studies reporting the fabrication of PHA composites and blends with various fillers: clay and its derivatives [25,26], plant fibers [27,28], lignin and holocellulose from lignocellulosic biowaste [29], wood shavings and sawdust, rice flour [30,31,32], etc. Such composites are promising materials for fabricating environmentally friendly and fully degradable products for various applications, and their importance cannot be overestimated.

Another direction of research is devoted to the potential uses of degradable polymers instead of toxic formaldehyde resins as binding material for plant wastes (sawdust, wood chips, etc.) to produce wood-plastic composites (WPCs) for construction and furniture industries. They are used to fabricate wood fiber and wood particle boards, sheets, etc. [33,34,35]. A number of studies address the properties of composites of several PHA types and wood flour, including their mechanical properties, moisture resistance, and degradability in natural media [36,37,38,39,40]. Being biodegradable and exhibiting hydrophobic, antioxidant, and gas barrier properties, PHAs can compete effectively with petroleum-based plastics on the packaging market [19,41]. Boxes, bottles, and bags made of PHAs and PHA blends with the synthetic polylactide or PHAs plasticized by natural materials (cellulose, starch, plant fibers, etc.) have been successfully tested as packaging materials for milk, vegetable oil, and farina and other free-flowing products [42,43,44,45,46].

The environmentally and socially significant PHA application is the construction of environmentally friendly pesticide formulations for protecting crops from weeds and pathogens, the so-called slow-release herbicide and fungicide formulations [47]. The newest line of research is the construction of pre-emergence pesticide formulations for soil application with controlled release of the active ingredient by embedding the active ingredients into biodegradable polymeric materials. The polymer matrix is degraded in soil by microflora to form harmless products, enabling the gradual and slow release of the active ingredients to soil and delivery of the pesticides to plants [48]. Such formulations are buried in soil simultaneously with seeds. Their advantages include their long action and reduction in the number of treatments of crops; longer activity of unstable pesticides; lower toxicity for biota and reduced accumulation in the food chain; conversion of the liquid forms to the solid ones, making them safe for use and simpler to transport [49,50]. The principal aspect of constructing slow-release pre-emergence pesticide formulations is the availability of appropriate materials that must have the following properties: controlled degradation in soil, environmental safety, and processability into granules, microcapsules, films, etc. by available methods compatible with pesticide production technologies. Although research on PHAs as material for embedding pesticides has been started rather recently, results have been obtained that suggest the high potential of these biopolymers for developing new-generation formulations for plant protection.

The slow-release formulations of pesticides embedded in PHA matrices described in the literature include the Sumilex and Ronilan fungicides [51]; herbicides ametryn, atrazine, metsulfuron-methyl, 2-methyl-4-chlorophenoxyacetic acid [52,53,54,55,56]. Jiangsu Changqing Agrochemical (China) developed and started the production of the highly effective fungicide—fenoxanil—loaded into P(3HB) microcapsules, which showed high parameters of controlled release and reduced environmental toxicity [57]. Recently, PHAs have been investigated as materials for manufacturing mulch films loaded with pesticides and intended for both suppressing weeds and preventing soil-transmitted plant root diseases [58,59]. Researchers of the Institute of Biophysics SB RAS (Russia) developed P(3HB)-based pre-emergence controlled release formulations of herbicides and fungicides (microparticles, films, pressed 3D forms, and granules) for soil application [60]. The authors of that book showed the high biological efficacy of the experimental formulations in controlling weeds and root rot pathogens in laboratory crops infested with weeds and infected by plant pathogens. Then, similar pesticide formulations were constructed by embedding herbicides and fungicides into blends of P(3HB) and natural materials (birch wood flour, peat, clay) [61,62]. The laboratory and micro-field experiments showed the high biological efficacy of the embedded pesticides in protecting cereal (wheat, barley) and vegetable (tomato, table beet) crops infested by weeds and infected by root rot pathogens [62,63,64,65,66,67,68,69,70,71,72].

In addition to cereal crops, a significant component of world food security is the potato, which is a most valuable farm crop, whose tubers are used for food, technical purposes, and animal feed. The significance of the potato is growing, as the productivity of cereal crops is decreasing everywhere. Potatoes are grown all over the world [73,74]. Potatoes are harvested from almost 20 million hectares, and the world potato production has exceeded 400 million tons [75]. Under optimal conditions, potato yield may potentially reach 60–100 t·ha^−1^, but the real yields are considerably lower because of the high incidence of diseases during the growing period and during storage. The data reported by FAO (Food and Agriculture Organization of the United Nations) show that annual global potato losses due to diseases reach 88.9 million tons amounting to US$3.4 billion, or 11.6% of the gross harvest; this is twice as much as the losses of cereal crops, vegetables, and sugar beet [74,76].

The decrease in potato productivity and saleable yield usually results from the damage caused by pests, weeds, and, especially, infections caused by microorganisms. As potatoes are grown under increasingly worse agrometeorological and phytosanitary conditions and tuber yields are becoming more and more unstable, there is a need for developing new protective measures in all potato-producing countries. Successful control of potato diseases should involve biological, chemical, and agricultural engineering measures. A modern trend in potato protection is to use environmentally safe crop protection products. However, chemical methods still prevail: pre-sowing treatment of planting stock with pesticides, spraying of the plants with pesticide solutions during the growing season (12 to 18 times per season), and pre-storage treatment of potato tubers. Yet, even when the plants are sprayed several times, potato yield losses can be rather substantial because of the low effectiveness of the fungicides, the use of heavily infected planting stock, and the emergence and development of plant pathogens resistant to the fungicides used. Many of the currently applied pesticides do not quite meet the requirements of both food safety and the safety of natural ecosystems and the biosphere. Chemical fungicides are nonspecific, kill non-target organisms, are often toxic to animals and humans, and cause the formation of resistant plant pathogen populations, which results in more frequent treatments and higher application rates of fungicides. The wide use of chemical pesticides pollutes the environment and causes environmental concerns. Because of these factors, along with the high cost of chemical protection of potatoes, the necessity arises to develop new and environmentally safe means and ways to reduce the pesticide impact on both potato crops and natural ecosystems and the entire environment.

The purpose of this study was to construct and characterize slow-release fungicide formulations for suppressing potato pathogens. The chemical structure, physicochemical properties, degradation behavior in soil, and biological activity of fungicides with different modes of action embedded in the degradable polymer/wood flour blend were studied for the first time.

## 2. Materials and Methods

### 2.1. Polymer

A degradable matrix for embedding fungicides was prepared from the most widely available and slowly degradable polyhydroxyalkanoate (PHA)—poly(3-hydroxybutyrate) homopolymer (P(3HB))—synthesized by using *Cupriavidus necator* B-10646 wild strain in accordance with the patented technology [77]. The polymer was extracted from cells with chloroform, and the extracts were precipitated using hexane. The process of polymer synthesis and analytical methods of polymer extraction and investigation of its properties were described in detail elsewhere [16].

### 2.2. Fungicides

Contact systemic fungicides with different modes of action were tested [78] (Xi’anTai Cheng Chem Co., Ltd., Yancheng, China) (Table 1):

### 2.3. Constructing Embedded Fungicide Formulations

P(3HB) samples with known properties were used to prepare blends with the natural material (birch wood flour) as the matrix for embedding fungicides. The polymer and the filler were pulverized by impact and shearing action in ultra-centrifugal mill ZM 200 (Retsch, Haan, Germany). The grinding of the components took place separately. To achieve high fineness of polymer grinding, the material and the mill housing with the grinding tools were preliminarily cooled at –80 °C for about 30 min in an Innova U101 freezer (New Brunswick Scientific, Enfield, CT, US). Then, a sample of polymer powder with a particle size of 200 µm was mixed with a wood flour sample with similar particle size and a fungicide. The fractional composition of the polymer and filler powders was determined using a vibratory sieve shaker AS 200 control (Retsch, Haan, Germany). Components were mixed in benchtop planetary mixer SpeedMixer DAC 250 SP (Hauschild Eng., Hamm, Germany); the blend period was 1 min, with the speed 1000 rpm, as described elsewhere [62]. The blends were processed by a spheronization technique using a granulator (Pharmag GmbH, Klipphausen, Germany), according to the developed and patented technology [62,63], to produce fungicide granules for protecting the potato from pathogens. The granules contained the components in the following ratios: P(3HB):wood flour:fungicide = 60:30:10 (when one fungicide was used) and 60:30:5:5 (wt.%) (when two fungicides were used). The granules were of two sizes: 1.5 mm and 3.0 mm in diameter and 4–5 mg and 8–10 mg in weight. They contained one of the four tested fungicides (azoxystrobin or difenoconazole, or mefenoxam, or prothioconazole) or two fungicides—azoxystrobin + mefenoxam.

### 2.4. Physico-Chemical Properties of Embedded Fungicides

The substances in the form of powders (poly(3-hydroxybutyrate), birch wood flour, initial fungicides) and three-component P(3HB)/wood flour/fungicide formulations were examined by using state-of-the-art physicochemical methods (SEM, IR spectroscopy, X-ray and thermal (DTA and DSC) analysis). The microstructure of the surface of granules was analyzed using scanning electron microscopy (a TM3000 Hitachi microscope with the QUANTAX 70 program, Tokyo, Japan). Prior to microscopy, the samples were sputter coated with platinum (at 25 mA, for 60 s), with an EM ACE200 (Leica, Vienna, Austria). The elemental composition of the samples was examined using scanning electron microscopy (a TM3000 Hitachi microscope with the QUANTAX 70 program, Tokyo, Japan). Thermal properties of the starting materials (polymer, birch wood flour, and fungicides) and the three-component formulations were analyzed using a DSC-1 differential scanning calorimeter (Mettler Toledo, Schwerzenbac, Switzerland). Melting points were determined from exothermal peaks in thermograms using the STARe software. Thermal degradation of the samples was investigated using a TGA2 thermal analysis system (Mettler Toledo, Schwerzenbac, Switzerland). X-ray structure analysis and determination of crystallinity of the samples were performed employing a D8ADVANCE X-ray powder diffractometer (Bruker AXS, Karlsruhe, Germany) (graphite monochromator in a reflected beam). Spectra were taken in a scan-step mode, with the scan step 0.04° and the 2-s exposure time to measure intensity at point (instrument mode—40 kW × 40 µA). IR spectra were taken in the 400–4000 cm^−1^ range using a “NICOLET 6700” FT-IR spectrometer (Thermo Scientific, Waltham, MA, USA) and a Smart Orbit accessory, by the attenuated total reflection (ATR) technique. The methods of analyses were described in detail elsewhere [62,63].

### 2.5. Degradation of Embedded Fungicides in Soil

Fungicide-loaded granules of two sizes were weighed, placed into nylon mesh bags, and buried in the field soil (200 g in 250-cm^3^ containers) at a depth of 2 cm. The granules were incubated in soil for 75 days at a temperature of 25 °C and a soil moisture content of 50%. The agro-transformed soil used in the experiment was a leached, heavy clay-loam, weakly structured chernozem. The soil was characterized by the high humus content (6.3%) and low nitrate nitrogen N-NO_3_ (4.7 mg·kg^−1^ soil) and low ammonium nitrogen N-NH4 (4.5 mg·kg^−1^ soil); it was high in available phosphorus and potassium (P_2_O_5_—269.2 mg·kg^−1^ soil; K_2_O—174.4 mg·kg^−1^ soil); the pH of soil solution was close to neutral (pH 7.3).

### 2.6. Chromatographic Analysis of Fungicide Concentrations in Soil

Soil fungicide concentrations were analyzed using high-performance liquid chromatography (HPLC), with an Agilent 1200 chromatographic system (Agilent Technologies, Santa Clara, CL, US) equipped with a diode matrix detector. An Eclipse XDB-C18 column was used. Fungicides were isolated from the soil as follows: 5–10 mL of water and 20–40 mL of acetonitrile or methanol (difenoconazole isolation) were added to 10–20 mg of dry soil and mixed using a Vortex mixer for 3–5 min. Then, 2–4 g sodium chloride and 1–2 g anhydrous magnesium sulfate were added, and the mixture was agitated for 1–3 min. Centrifugation was performed for 5 min at 3000 g. Then, acetonitrile was removed by using a rotary evaporator, under vacuum, at 40 °C. Acetonitrile was added to measure azoxystrobin, prothioconazole, and mefenoxam; methanol was added to measure difenoconazole.

### 2.7. A Microbiological Study

The structure of the soil microbial community in laboratory microecosystems was analyzed by using generally accepted microbiological methods. Changes in the abundances of the ecological-trophic groups of bacteria were monitored by plating soil suspensions (104–106 dilutions) on nutrient media. Copiotrophic bacteria were isolated on meat peptone agar, prototrophs—on starch and ammonia agar, oligotrophs—on soil extract agar, and nitrogen-fixing bacteria—on Ashby’s medium [79]. The plates were incubated in the temperature-controlled cabinet at 30 °C for 5–7 d. Mineralization coefficients (Cmin) and oligotrophy coefficients (Colig) were determined as the ratio of prototrophic to copiotrophic microorganisms and the ratio of oligotrophic to copiotrophic microorganisms, respectively. Dominant bacterial species were identified by the Matrix Assisted Laser Desorption/Ionization Time-of-Flight (MALDI-TOF) mass spectrometry method, performed using MALDI-TOF MS Microflex (BrukerDaltonics, Bremen, Germany). Counting the total number of microfungi in soil samples was carried out by plating soil suspension on potato-dextrose agar with benzylpenicillin (1,000,000 U⋅L^−1^ of medium) to suppress the growth of bacteria. All platings were done in triplicate from dilutions to 104. The plates were incubated in the temperature-controlled cabinet at 25 °C for 7–10 days. Microscopic examination of colonies was performed using an AxioStar microscope (Carl Zeiss MicroImaging GmbH, Göttingen, Germany). Microfungi were identified by cultural and morphological features according to the identification guides [80].

### 2.8. Testing Biological Activity of Embedded Fungicides

The biological activity of embedded fungicides was tested in vitro, in cultures of phytopathogenic fungi, using experimental granules. In the positive control, free fungicides at the same concentrations were used. The following species of phytopathogenic fungi were tested: Alternaria alternata, Alternaria longipes, Boeremia exigua, Phytophthora infestans, Rhizoctonia solani, Fusarium oxysporum, Fusarium redolens, Fusarium solani, Fusarium equiseti, and Fusarium vanettenii. These pathogens were previously isolated from the samples of the study field soil and tubers of potato cv. Krasnoyarskiy ranniy and identified using conventional culture-based and molecular-genetic methods. The nucleotide sequences of the 28S rRNA gene fragments of the isolated fungi are available in GenBank (No. MZ424190-MZ424199) [81].

The fungistatic activity of the embedded fungicides was investigated by diffusion method on the wort agar and compared to the positive control (with the free fungicides placed on the Petri dish at the same concentration as in the treatment) and the negative control (with no fungicides). The various species of microfungi causing common potato diseases were grown in lawn plates on potato-dextrose agar (PDA, HiMedia, Thane, India) at a temperature of 25 °C for 5–7 d. Agar blocks with the colonies showing the best growth were cut out under aseptic conditions. Then, in a Petri dish with sterile wort agar medium, a block with mycelium and one of the tested fungicide forms were placed onto the agar on the opposite sides of the dish. The dishes were incubated for 7–10 d in a temperature-controlled cabinet at 25 °C. To assess the effectiveness of the experimental granules with fungicides, the diameters of the colonies in the treatments were compared to those in the positive and negative controls. Each procedure was performed in triplicate, and the results were photographed. 

### 2.9. Statistics

Statistical analysis of the results was performed by conventional methods, using the standard software package of Microsoft Excel. Arithmetic means and standard deviations were found. The statistical significance of results was determined using the Mann–Whitney test (significance level: *p* < 0.05). Statistical analysis of the surface properties of the samples was performed by using embedded methods of the DSA-4 software.

## 3. Results and Discussion

### 3.1. Characterization of Fungicides

Systemic contact fungicides with different modes of action were used in the present study [78]. Azoxystrobin inhibits mitochondrial respiration in pathogen cells by blocking electron transport in the cytochrome b and c1 chain; it has a lasting protective effect, protecting plant roots and aboveground parts. Difenoconazole destroys the membrane function of the cells of pathogens. Mefenoxam exhibits eradication and protective activities; it inhibits protein formation in fungi, inhibiting the synthesis of ribosomal RNA. Prothioconazole inhibits C-14-demethylase, which takes part in the biosynthesis of fungal sterols; it exhibits protective, curative, and eradication activities; in the plant, it is metabolized to form a more stable compound—prothioconazole-desthio.

**Figure 1:**
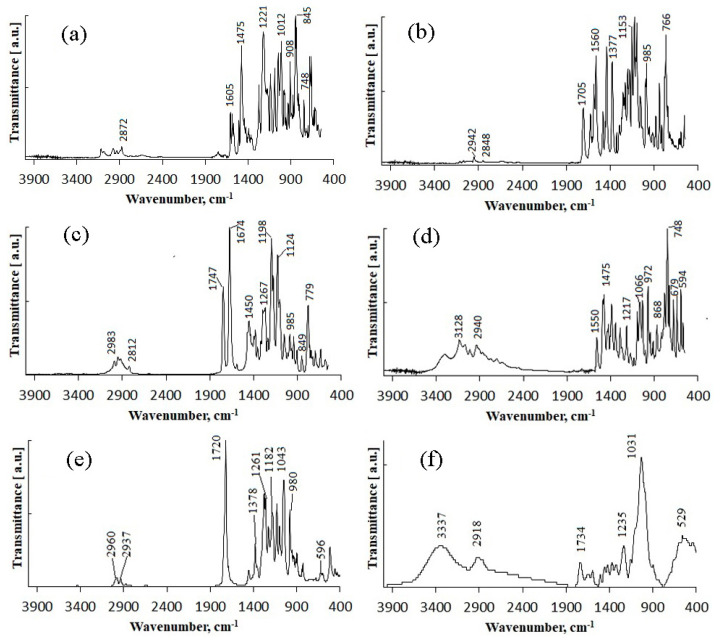
IR spectra of initial matrix materials and fungicides: (**a**)—difenoconazole; (**b**)—azoxystrobin; (**c**)—mefenoxam; (**d**)—prothioconazole; (**e**)—P(3HB); (**f**)—birch wood flour.

The tested fungicides are complex compounds combining various classes of substances and having a complex spectrum in the infrared region. IR spectra of the initial fungicides are shown in Figure 1. 

Difenoconazole is an aromatic ester, dioxolane, triazole, cyclic ketal. The dioxolane ring had 702; 723; 748 cm^−1^ absorption bands, corresponding to stretching vibrations of the C=C bond. The bands of various intensities—908, 930, 1012 cm^−1^—corresponded to the C-O-C stretching vibrations in dioxolane. The strong-intensity absorption band at 1220 cm^−1^ corresponded to the C-O-C stretching vibrations in the phenyl group, and the 1600–1575 cm^−1^ bands of varying intensity were assigned to pulses of the carbon skeleton in the phenyl group. The strong-intensity absorption band at 844 cm^−1^ and the medium-intensity band at 905 cm^−1^ were assigned to out-of-plane deformation vibrations of C-H groups in the aromatic ring. The absorption band at 1475 cm^−1^ was assigned to stretching vibrations of the C=N groups of the molecule triazole part; that band was overlapped by the absorption band of stretching vibrations of the aromatic ring in the 1440—1456 cm^−1^ range. The medium-intensity bands at 933—1138 cm^−1^ were assigned to deformation vibrations of C-H groups of the aromatic ring. The weak-intensity band at 2928 cm^−1^ was assigned to stretching vibrations of methyl groups. 

Azoxystrobin is a nitrile, (aryloxy)pyrimidine, enol ether, and methyl ether. It had the medium-intensity absorption band at 877 cm^−1^, which was assigned to deformation vibrations of C-H groups in the pyrimidine ring. Vibrations of the pyrimidine ring had the medium-intensity absorption band at 1585 cm^−1^. For methoxy groups, weak stretching vibrations were recorded at 2845 cm^−1^. The broad, strong-intensity absorption band in the 1050–1150 cm^−1^ range was assigned to stretching vibrations of the C-O-C ether group. The absorption bands of varying intensity at 766 cm^−1^ and 720 cm^−1^ were assigned to out-of-plane deformation vibrations of C-H groups in the phenyl ring, typical in the presence of substituents at positions 1–2. In the 1440–1456 cm^−1^ range, the bands of varying intensity corresponded to stretching vibrations of the aromatic ring. Deformation vibrations of the C-H bonds in the vinyl substituent in the trans form had the following absorption bands: in-plane at 1290 cm^−1^ and out-of-plane at 985 cm^−1^. Stretching vibrations of the carbonyl group were at 1705 cm^−1^. This fungicide was previously studied in [62]; the other fungicides were studied in the present work for the first time. 

Mefenoxam is an alanine derivative, aromatic amide, carboxamide, ether, and methyl ether. The aromatic ring had weak-intensity absorption bands at 941, 985, and 1049 cm^−1^, which corresponded to deformation in-plane vibrations of the C-H groups, and at 904 and 849 cm^−1^, which corresponded to deformation out-of-plane vibrations of the C-H groups. The absorption band at 1450 cm^−1^ was assigned to stretching vibrations of the aromatic ring; it was partly overlapped by the band of deformation vibrations of CH_2_ groups. The weak-intensity band at 2812 cm^−1^ and the strong-intensity band at 1747 cm^−1^ were assigned to stretching vibrations of the carbonyl group. The medium-intensity absorption band at 1124 cm^−1^ corresponded to symmetric stretching vibrations of C-O- in the ester group. The strong-intensity absorption band at 1198 cm^−1^ corresponded to asymmetric stretching vibrations of C-O- in the ester group. The strong-intensity absorption band at 1674 cm^−1^ was assigned to the tertiary amide. 

Prothioconazole is a monochlorobenzene, triazole, tertiary alcohol, cyclopropane, and thiocarbonyl compound. The medium-intensity absorption band at 1217 cm^−1^ corresponded to stretching vibrations of the triazole ring C=N groups. Absorption bands at 727 and 779 cm^−1^ were assigned to out-of-plane deformation vibrations of C-H groups in the phenyl ring, typical in the presence of substituents at positions 1–2. The absorption bands at 947 and 1012 cm^−1^ corresponded to in-plane deformation vibrations of C-H groups. The strong-intensity band at 746 cm^−1^ was assigned to stretching vibrations of the C-Cl bond. The broad split absorption band in the 1018–1117 cm^−1^ range was assigned to stretching vibrations of the (C-O-) group. The absorption band at 1290 cm^−1^ corresponded to deformation vibrations of C-H in phenyl that had a substituent at para-position. The region of absorption of the N-H stretching vibrations in a non-associated state at 3138 cm^−1^ was overlapped by the broad medium-intensity band at 3400–3200 cm^−1^ corresponding to stretching vibrations of intra- and inter-molecular associated hydrogens in the hydroxyl group. 

IR spectra of materials used to construct the matrix for embedding the fungicides are also shown in Figure 1. The IR absorption spectra of the degradable polymer P(3HB) contained absorption bands corresponding to vibrations of the main structural components of polymers except for the absorption bands of vibrations of the terminal C-OH and COOH groups. The bands of the ordered optical densities (crystalline phase) were in the 1228 cm^−1^ range while the bands of the amorphous phase were shifted to 1182 cm^−1^. This is consistent with the data reported in other studies [82,83,84]. The spectra show distinct absorption bands of asymmetric stretching of CH_3_- and CH_2_-groups (2978 and 2994 cm^−1^); symmetric stretching of CH- and CH_2_ groups (2994 and 2934 cm^−1^); stretching vibrations of conjugated (1687 cm^−1^) and unconjugated (1720 cm^−1^) carbonyl groups C=O; skeletal CH vibrations (599 cm^−1^); and CH bending (622 cm^−1^). PHAs are hydrophobic compounds, in which molecules of water interact with the hydrogen atom of the methyl group or with the oxygen atom of the carboxyl group through hydrogen bonding. The narrow bands at 3436 cm^−1^ are typical of the hydrogen-bonded OH group. The 1687 cm^−1^ band is characteristic of hydrogen vibration in the OH group interacting with the oxygen atom through hydrogen bonding. The 2874-, 2934-, 2978-, and 2994-cm^−1^ absorption bands are typical of the bonded OH group and may be a component of the COOH dimer group. In the 3000–2500 cm^−1^ range, there is a group of weak bands typical of the dimers of carboxylic acids.

IR spectra of birch wood flour show in-plane deformation vibrations of the guaiacyl ring of lignin of leaves (1031 cm^−1^), stretching of -C-O-;-C-O-C- (1235 cm^−1^), skeletal vibrations of the syringyl ring of lignin of leaves (1324 cm^−1^), stretching and skeletal vibrations of benzene ring (1422 cm^−1^, 1504 cm^−1^, 1593 cm^−1^), stretching of -C=O of conjugated and unconjugated groups (1652 cm^−1^, 1734 cm^−1^), and intramolecular and intermolecular stretching of -OH groups and water (3337–3342 cm^−1^).

**Figure 2:**
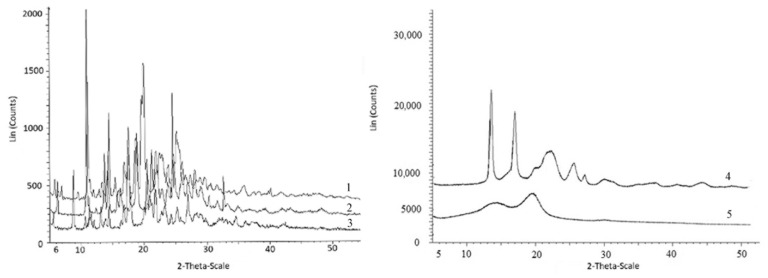
X-ray spectra of initial matrix materials and fungicides: 1—difenoconazole; 2—azoxystrobin; 3—prothioconazole and materials of the matrix 4—P(3HB) and 5—birch wood flour.

**Figure 3:**
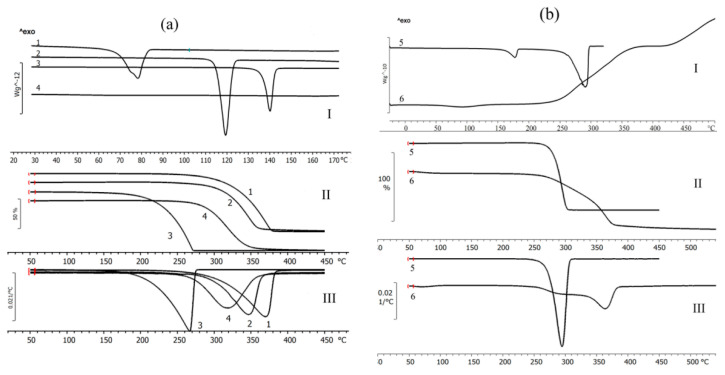
Results of thermal analysis of fungicides (**a**) and materials of the matrix (polymer and wood flour) (**b**): I—DSC curves; II—TGA curves; III—weight loss of the samples under heating in differential form: 1—difenoconazole; 2—azoxystrobin; 3—mefenoxam; 4—prothioconazole; 5—P(3HB); 6—birch wood flour.

Physico-chemical properties of fungicides and materials used to construct matrix for embedding them are shown in Table 2, Figure 2 and Figure 3.

The fungicides tested in this study had similar degrees of crystallinity; their thermal parameters were different. The properties of the fungicides differed from the properties of the matrix materials. P(3HB) is a highly crystalline material, with a crystalline phase; the degree of crystallinity (C_x_) of the P(3HB) sample used in this study was 76%. Diffraction peaks in 2 θ = 13.4, 16.8, 20, 22.2, and 25.5° had a similar pattern when compared with previous crystallographic data for this polymer [85,86]. The degree of crystallinity of birch wood flour was considerably lower (26%); X-ray images showed diffraction peaks in 2 θ = 14.2, 19.6, and 25.5° (Figure 2). The degrees of crystallinity of the fungicide powders (difenoconazole, azoxystrobin, mefenoxam, and prothioconazole) varied between 56 and 66% but were comparable (Figure 2). Mefenoxam is a viscous liquid, and, thus, no X-ray diffraction analysis could be performed on it.

Thermal properties of the fungicides, polymer, and wood flour were examined using DSC and TGA (Figure 3). Thermograms of the polymer showed the peak of the melting temperature; P(3HB) is a thermoplastic material with a distinct difference between melting point (176 °C) and temperature for thermal degradation (287 °C). The crystallization temperature of the polymer is around 108 °C and melting enthalpy is 89.3 J·g^−1^. Birch wood flour (filler) is not a thermoplastic material. The thermogram of wood flour showed a small peak at a temperature between 70 and 130 °C, which was associated with the evaporation of moisture and volatile organic compounds. The thermal decomposition of wood flour begins at 220 °C.

The thermal properties of fungicides determined in this study, such as T_melt_, (Figure 3), differed somewhat from the data presented in reference materials [87,88]. That was probably caused by the differences in the origin and degree of purity of the specimens. For example, the melting point of azoxystrobin was 118 °C while according to the literature data, azoxystrobin melts at 166 °C. Based on results of DSC, the melting temperature of mefenoxam was 140 °C, but the literature data on the melting point of this fungicide are contradictory, reporting values between 38.7 and 72 °C. The melting temperature of prothioconazole reported in the literature is 142 °C, although the specimen examined in the present study had no melting point (Figure 3).

Results of TGA indicate rather high thermal stability of fungicides. Prothioconazole had the lowest and difenoconazole the highest thermal degradation temperature (236 °C and 329 °C, respectively) (Figure 3). All fungicides had rather wide temperature ranges within which thermal degradation occurred. The ranges of thermal degradation temperatures of all fungicides overlapped the ranges of thermal degradation temperatures of P(3HB) and wood flour.

The fungicides, polymer, and birch wood flour were homogenized, and experimental formulations were prepared. Polymer, wood flour, and fungicide were mixed in preset proportions in a planetary mixer to fabricate fungicide formulations. 

### 3.2. Characterization of Embedded Fungicides

**Figure 4:**
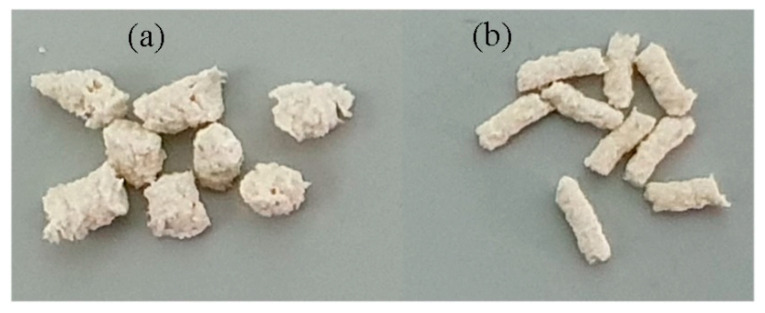
Photographs of fungicide formulations in the form of 1.5-mm (**a**) and 3.0-mm (**b**) granules.

**Figure 5:**
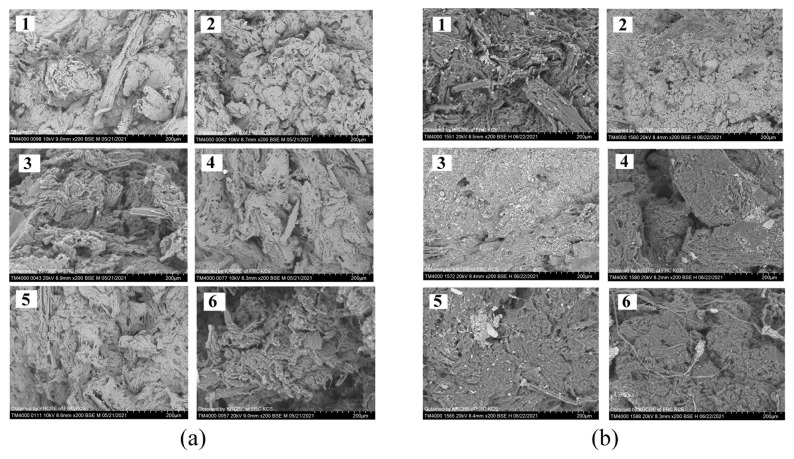
SEM images of the microstructure of fungicide loaded granules before soil application (**a**) and during degradation in soil (**b**): 1—P(3HB)/wood flour/difenoconazole; 2—P(3HB)/wood flour/azoxystrobin; 3—P(3HB)/wood flour/mefenoxam; 4—P(3HB)/wood flour/azoxystrobin + mefenoxam; 5—P(3HB)/wood flour/prothioconazole; 6—P(3HB)/wood flour.

Photographs and SEM images of fungicide-loaded granules are presented in Figure 4 and Figure 5. Differences in the polymer and wood flour properties became noticeable as differences in the surface structure of the granules (Figure 5). SEM images of the granule surfaces show that wood flour used as a component of the blend altered the surface microstructure: the surface was rough, and oblong wood flour particles were visible. Azoxystrobin mixed with P(3HB) and natural fillers was studied previously [62]; the other fungicides embedded in the degradable P(3HB)/birch wood flour blend were studied for the first time in the present work. 

**Figure 6:**
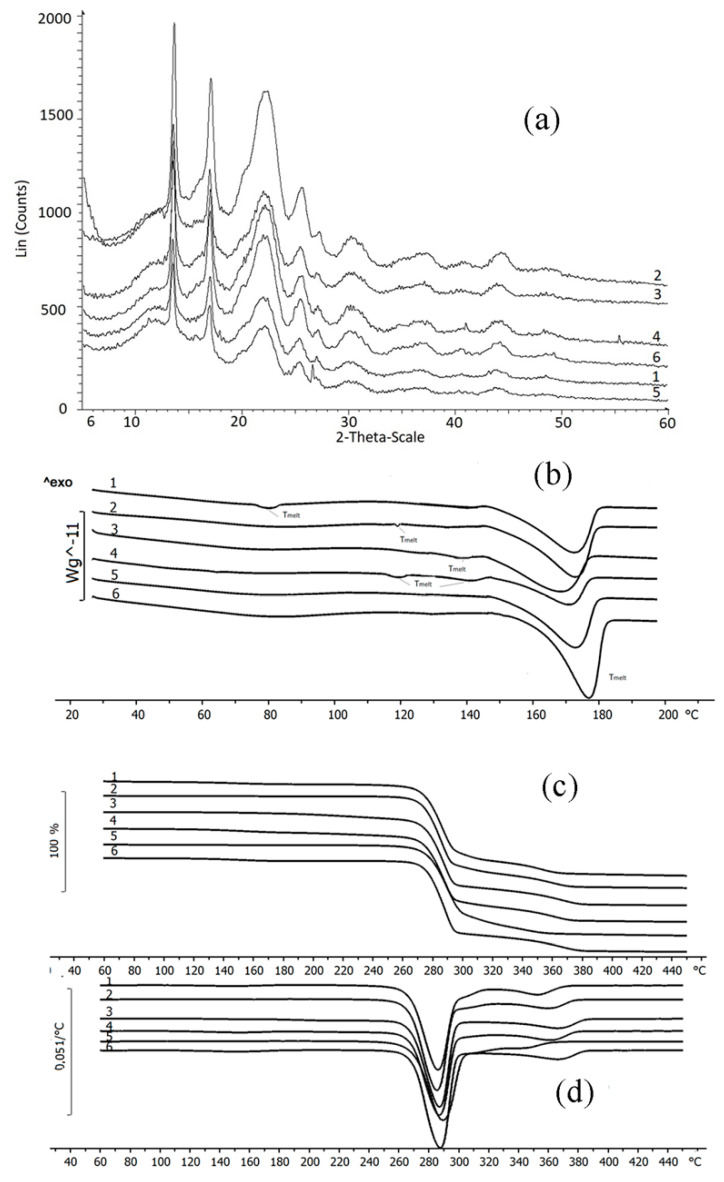
X-ray images (**a**), DSC curves (**b**), DTA curves (**c**) and weight loss of the fungicide loaded granules in the differential form (**d**): 1—P(3HB)/wood flour/difenoconazole; 2—P(3HB)/wood flour/azoxystrobin; 3—P(3HB)/wood flour/mefenoxam; 4—P(3HB)/wood flour/azoxystrobin + mefenoxam; 5—P(3HB)/wood flour/prothioconazole; 6—P(3HB)/wood flour.

The properties and interactions between fungicides and degradable P(3HB)/wood flour matrix were studied using X-ray and thermal analyses and IR spectroscopy. Filling of P(3HB) with wood flour did not affect thermal properties but caused amorphization of the polymer: the degree of crystallinity of the polymer/wood flour blend dropped to 58% (Table 2). X-ray images of fungicide formulations were a sum of diffraction peaks of their components (polymer, wood flour, and fungicides) (Figure 6). The filling of the P(3HB)/wood flour matrix, whose Cx was 58%, with different fungicides resulted in a decrease in the degree of crystallinity; the most considerable decrease was observed in difenoconazole, mefenoxam, and prothioconazole formulations: the Cx of these granules was 42–43%. The change in crystallization kinetics of the polymer blended with wood flour was caused by the use of a rather high percentage of the filler (30%). This must have decreased the free volume necessary for nucleation of spherulites to occur, preventing the development of the crystalline phase and, thus, reducing it and increasing the amorphous phase.

Thermograms of the fungicide formulations were taken in a wide range of temperatures including boundaries of melting and thermal decomposition (Figure 6). DSC was used as one of the most informative methods for determining the thermal properties of polymers and polymer-based blends, as the melting behavior of components of the blend provides evidence of the degree of miscibility and interactions of the components. All thermograms contained melting peaks corresponding to P(3HB) and fungicides; melting temperatures and enthalpies are listed in Table 2. Results showed that the fillers (30% *w·w*^−1^) and fungicides (10% *w·w*^−1^) did not significantly affect the temperatures of melting and thermal decomposition of P(3HB) but considerably decreased enthalpy of melting. That could indicate a decrease in the size of crystals and an increase in the percentage of defects in them, resulting in a reduction in the amount of energy necessary for the crystals to melt. Compared to the enthalpy of the initial P(3HB) (89.1 J·g^−1^), the specimens containing 30% wood flour and difenoconazole showed the smallest decrease in enthalpy (to 51.7 J·g^−1^) and the granules containing azoxystrobin + mefenoxam showed the greatest decrease in enthalpy (to 29.2 J·g^−1^).

As the components of the composites (polymer, wood flour, and fungicide) were mixed mechanically, the chemical structure of the polymer remained unchanged, and no considerable effect on the melting temperature was observed; the melting peak was shifted by 3–4 °C for granules loaded with different fungicides. A certain shift in the melting range of the granules could be caused by a change in the thermodynamic properties of the polymer/wood flour/fungicide system. The melting point of each study fungicide was lower than that of P(3HB), and, thus, there was a liquid phase in the system, which resulted in a change in heat transfer. 

TGA curves had two degradation regions for each sample because the ranges of degradation temperatures of the components overlapped. P(3HB) lost 98% of its mass at temperatures between 275 °C and 293 °C; wood flour was degraded at a wide temperature range, between 250 °C and 500 °C; fungicide degradation temperatures varied between 236 °C (prothioconazole) and 329 °C (difenoconazole). Each sample lost 68–70% of its mass at temperatures between 273 °C and 293 °C, which corresponded to the thermal degradation of P(3HB) and partial degradation of birch wood flour. In the second phase, the degradation of wood flour and fungicides continued.

Filling of P(3HB) with birch wood flour resulted in a decrease in P(3HB) crystallization temperature from 108 °C to 91 °C. Wood flour particles could serve as defect centers and limit the space for the crystallization of polymer molecules, resulting in the production of smaller crystals and causing the temperature of crystallization to decrease. The presence of pesticides in the granules further reduced the temperature of crystallization, and the most considerable reduction in crystallization temperature was noted for the samples containing mefenoxam. P(3HB)/wood flour/mefenoxam samples were crystallized at 70 °C, P(3HB)/wood flour/azoxystrobin + mefenoxam at 79 °C, P(3HB)/wood flour/prothioconazole and P(3HB)/wood flour/difenoconazole samples at 81 °C, and the P(3HB)/wood flour/azoxystrobin sample was crystallized at the highest temperature—88 °C. The temperature range at which P(3HB) was crystallized increased. 

Considerable research effort has been devoted recently to the so-called green composites, made of PHA polymers and various natural materials. In a study by Melendez-Rodriguez et al. (2019) [32], films fabricated by thermo-compression at 180 °C from P(3HB) and P(3HB-co-3HV) melt filled with rice husk flour had lower crystallinity compared to the initial polymers, remained thermostable, and had improved mechanical plasticity. Filling of P(3HB) with lignocellulose enhanced thermostability of the melt of that blend; DSC showed that the filler facilitated crystallization of the polymer melt and potentially enabled controlling the physical aging of P(3HB) [29].

**Figure 7:**
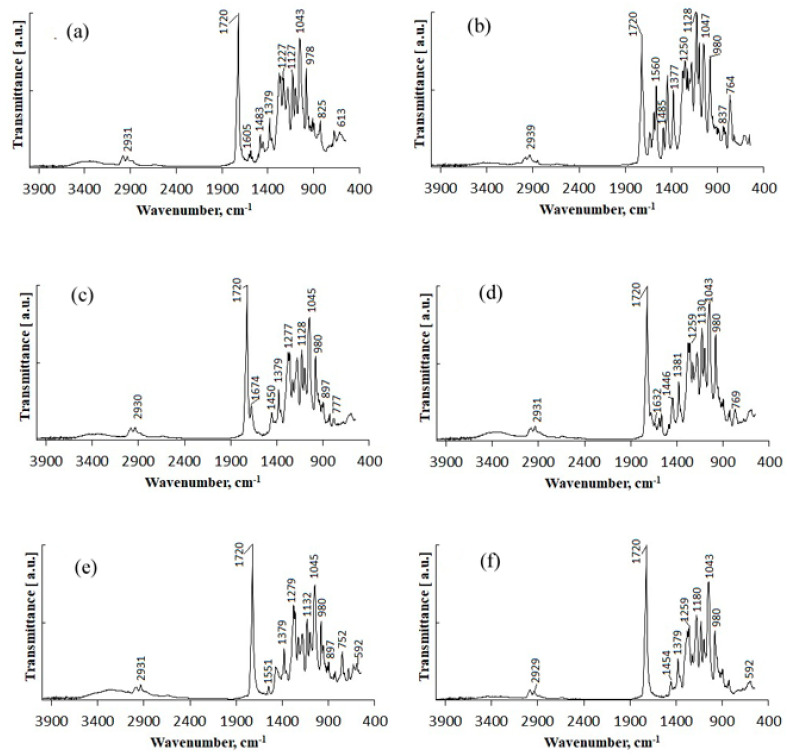
IR spectra of fungicide formulations: (**a**)—P(3HB)/wood flour/difenoconazole; (**b**)—P(3HB)/wood flour/azoxystrobin; (**c**)—P(3HB)/wood flour/mefenoxam; (**d**)—P(3HB)/wood flour/azoxystrobin + mefenoxam; (**e**)—P(3HB)/wood flour/prothioconazole; (**f**)—P(3HB)/wood flour.

IR spectroscopy was used to reveal possible structural changes in the polymer/wood flour/fungicide formulations. The intensity of bands in the low-frequency region can provide data not only on the crystalline to amorphous phase ratio in the blends but also on the type of interactions of components of the blends. The IR spectra of the fungicide formulations (Figure 7) did not show any new absorption bands or any considerable shifts of the bands typical for the components of the matrix (polymer and birch wood flour) and fungicides. The IR spectrum contained absorption bands typical of the tested fungicides. The IR spectra clearly showed characteristic stretching vibrations of the C=O carbonyl groups of P(3HB) (1700–1760 cm^−1^) and asymmetric stretching vibrations of CH_3_- and CH_2_- groups (2994, 2974, 2936 cm^−1^) with overlapping bands typical of fungicides. For wood flour, stretching and skeletal vibrations of the benzene ring (1422 cm^−1^, 1504 cm^−1^, 1593 cm^−1^) were retained (Figure 7f).

The IR spectra of P(3HB)/wood flour/difenoconazole granules contained absorption bands at 598; 622 cm^−1^, which corresponded to the C-H skeletal and deformation vibrations. Stretching vibrations of the C=C bond in the dioxolane ring of the difenoconazole molecule had weak-intensity absorption bands at 696; 702; 750 cm^−1^. Absorption bands of out-of-plane deformation vibrations of the C-H groups in the aromatic ring were shifted to 825 cm^−1^ and 901 cm^−1^. The C-O-C absorptions band overlapped the absorption band of C-H bending in the guaiacyl ring of lignin and degenerated into a small shoulder at 1002 cm^−1^. The spectrum also contained pure absorption bands corresponding to difenoconazole, indicating that no chemical change had occurred in the molecule. The strong-intensity absorption band of C=N stretching vibrations in the difenoconazole triazole part at 1475 cm^−1^ was scaled up to a medium-intensity absorption band and shifted to 1493 cm^−1^. The broad split medium-intensity peak at 1575–1605 cm^−1^ corresponded to pulses of the carbon skeleton in the phenyl group of difenoconazole. Absorption bands of stretching vibrations of ether bonds (-C-O-C-) of the components of the granules overlapped and shifted to the long-wavelength part of the spectrum, to 1236 cm^−1^; pure difenoconazole had the strong-intensity peak at 1225 cm^−1^, and the P(3HB)/wood flour blend had the weak-intensity peak at 1228 cm^−1^. At 1720 cm^−1^, there was a strong-intensity absorption band corresponding to stretching vibrations of the non-conjugated -C=O group in the P(3HB) molecule. The broad absorption band at 2862–3004 cm^−1^ corresponded to symmetric and asymmetric stretching vibrations of -CH; -CH_2_-; -CH_3_. The spectrum of the granules did not contain an absorption band of stretching vibrations associated with O-H at 3050–3128 cm^−1^, in contrast to the spectrum of pure difenoconazole. However, in the spectrum of the granules, the absorption band in the 3200–3550 cm^−1^ range, where there were absorption bands of stretching vibrations of non-conjugated O-H groups, had a larger area and stronger intensity, which might indicate that no hydrogen bonds existed between components of the mixture.

The spectrum of the P(3HB)/wood flour/azoxystrobin granules also contained absorption bands typical of the individual components of the mixture. The absorption band corresponding to the out-of-plane deformation vibrations of C-H groups in the phenyl ring that had substituents at position 1,2, typical of azoxystrobin, was shifted from 766 cm^−1^ to 764 cm^−1^. The region of deformation vibrations of the C-H group of the azoxystrobin pyrimidine ring overlapped the region of deformation vibrations of -CH_2_; -CH_3_ of P(3HB)/wood flour at 837 cm^−1^; 841 cm^−1^. The region of absorption of deformation vibrations of the double-bonded C-H groups that were in trans position of azoxystrobin (945 cm^−1^) overlapped the absorption band of out-of-plane deformation vibrations of -CH groups of P(3HB) at 980 cm^−1^. Other changes included stronger intensity of absorption bands characterizing the ether bond (-C-O-C-); a split band with two peaks at 1047; 1055 cm^−1^ corresponding to stretching vibrations; a broad absorption band in the 1093–1167 cm^−1^ range with two peaks at 1095 cm^−1^ and 1124 cm^−1^, corresponding to symmetric stretching vibrations at 1227 cm^−1^; at 1250 cm^−1^, corresponding to asymmetric stretching vibrations. Absorption bands corresponding to the pyrimidine ring of azoxystrobin were located in the 1450–1653 cm^−1^ range. The medium-intensity absorption band at 1485 cm^−1^ was assigned to stretching vibrations of the C=N group in the azoxystrobin pyrimidine component; the absorption bands at 1560; 1585 cm^−1^ corresponded to vibrations of the pyrimidine ring. The medium-intensity absorption band at 1624 cm^−1^ corresponded to deformation vibrations of C-N. A strong-intensity absorption band at 1720 cm^−1^ corresponded to stretching vibrations of the non-conjugated -C=O group in the P(3HB) molecule. No changes were observed in the intensity and type of absorption bands in the 3200–3550 cm^−1^ range, suggesting that there were no intra- and intermolecular hydrogen bonds.

The absorption spectrum of the P(3HB)/wood flour/mefenoxam granules also contained bands typical of the components of the mixture. The weak-intensity band at 769 cm^−1^ corresponded to deformation vibrations of the C-N bond in mefenoxam. The absorption band of stretching vibrations of tertiary amide (1674 cm^−1^) overlapped the strong-intensity band of deformation vibrations of the non-associated carbonyl group at 1720 cm^−1^. Other changes in the spectrum included stronger intensity of absorption bands characterizing the ether bond, stretching, and deformation vibrations of -CH; -CH_2_; CH_3_. No changes were observed in the intensity and position of the bands in the 3200–3500 cm^−1^ range, characterizing intra- and intermolecular hydrogen interactions. 

In the absorption spectra of the granules containing two fungicides—azoxystrobin + mefenoxam—there were absorption bands corresponding to each fungicide, that is, both were present in the granules. Deformation vibrations of the C-N bond in mefenoxam were shifted to the long-wavelength part of the spectrum, to 766 cm^−1^. The medium-intensity absorption band assigned to stretching vibrations of the C=N group in the azoxystrobin pyrimidine component was shifted to 1637 cm^−1^. The absorption spectrum of the granules was a combination of the absorption spectra of their components. There were no new absorption bands, which would suggest the formation of new chemical bonds

Prothioconazole in the absorption spectrum of the granules had an absorption band at 592 cm^−1^—skeletal and deformation vibrations of the carbon-carbon bond; a set of absorption bands of varying intensity at 627; 669; 725; 752 cm^−1^, which corresponded to deformation vibrations of monochlorobenzene; an absorption band of stretching vibrations of monochlorobenzene C-Cl at 752 cm^−1^; the weak-intensity band was shifted from 868 cm^−1^ to 897 cm^−1^. The absorption band of deformation vibrations of the C=N bond in triazole overlapped deformation vibrations of methyl groups at 1458 cm^−1^. The spectrum also contained a weak-intensity absorption band at 1537 cm^−1^, which was typical of stretching vibrations of the prothioconazole alcohol group. In the 3000–3500 cm^−1^ range, stretching vibrations of -O-H and -N-H groups overlapped. The absence of the absorption bands at 3128; 3307 cm^−1^ might indicate that the -N-H groups of prothioconazole were involved in the formation of intra- and intermolecular hydrogen bonds.

The present study, for the first time, demonstrated that no chemical bonds were established between components of the polymer-based [(P(3HB)] matrix and fungicides embedded in it. That is, experimental pesticide formulations were physical mixtures of their components, and there was no significant interplay between the polymer and embedded fungicides. A similar result, showing that no chemical bonds were established between P(3HB) and natural materials in the blend, was reported in a study by Thomas et al. (2019) [62]; peat, clay, and wood flour were tested as fillers. The same data were obtained in studies of P(3HB)/natural material/pesticide formulations prepared by cold compaction and granules prepared from paste-like blends using a spheronization technique. The metribuzin, tribenuron-methyl, and fenoxaprop-P-ethyl herbicides [63] and the azoxystrobin, epoxiconazole, and tebuconazole fungicides [65] loaded into the degradable polymer/natural material matrix were found to be physical mixtures: IR spectroscopy did not reveal any new chemical bonds between P(3HB) and any of the pesticides. When, however, composites containing PHAs were prepared using another method, a different result was obtained. A study of the composite of sepiolite nanoclay and P(3HB-co-4HB) fabricated by melt compounding showed that during the thermal extrusion process, the reactive coupling agent successfully established strong chemical ‘‘bridges” between the biopolymer and sepiolite by Si-O-C bonds. As a result, it generated a new hybrid nanostructure, that is, a sepiolite-grafted P(3HB-co-4HB)(sepiolite-g-P(3HB-co-4HB)) nanocomposite [26]. Thus, the technique employed to process blends of PHA polymers and natural materials influences the chemical interactions in such blends.

A search of the literature shows that rather much research has been recently devoted to the development of new fungicide formulations with the active ingredient embedded in various degradable materials [49,89]. Recent years have seen the publication of studies on micro- and nano-sized fungicide formulations used as sprays to protect potato and other vegetable crops from pathogenic fungi including Fusarium and Phytophthora. Several studies reported encapsulation of the azoxystrobin fungicide in silica, chitosan, and poly(lactide/glycolide) microparticles to produce slow-release formulations [90,91,92]. The pyraclostrobin fungicide was encapsulated in polylactide/chitosan microparticles [93,94]; carbendazim was loaded into nanospheres of polylactic acid and poly(lactide/glycolide) [95]. Pesticides in the form of microparticle or microcapsule suspensions or emulsions have a number of disadvantages: complex composition, multistage and complicated fabrication, and potential hazard to beneficial biota.

The data on the newest and most promising approach—fabrication and pre-emergence application of slow-release targeted pesticide formulations—are highly limited. Only a few studies report on the production of embedded fungicides for soil application. The thiram fungicide was entrapped in agar and alginate matrices, and its release kinetics to water and soil was described [96]; in another study, thiram was incorporated in nonwoven polylactic acid fibers [97].

A fungicide formulation based on zinc and manganese ions was prepared in the form of nanoparticles coated by polyethylene glycol; the formulation was tested against mildew and Pythiaceae fungi [98]. Controlled-release nano-formulations of mancozeb based on polyethylene glycol were described in a study by Majumder et al. (2016) [99]. Other authors [100] reported loading bioactive eugenol preparations into the chitosan-based matrix and producing formulations that were used to treat potatoes, effectively controlling potato blight. Researchers of Moscow State University (Russia) produced a series of the Quadris fungicide formulations (with azoxystrobin as the active ingredient) based on polyacrylate gels to be used as a pre-emergence fungicide [101,102]; the formulations were tested in the field and found to effectively control potato diseases.

### 3.3. Degradation Behavior of Fungicide Formulations in Soil and Release Kinetics of Active Ingredients 

The fungicide formulations developed in the present study are intended for soil application, and, thus, their degradation behavior in soil is the most significant issue. The degradation behavior of the polymer matrix determines the period during which the formulations will function in soil, release kinetics of the active ingredients, and fungicide concentrations in soil. In turn, the degradation of PHAs, which are used as the matrix for loading fungicides, is determined by various factors including the chemical composition and structure of the PHA used, the microbial component of the soil (the main agent of biodegradation of the polymer matrix), and weather conditions. 

Microbiological degradation of the fungicide formulations was studied in laboratory microcosms with agro-transformed pre-characterized soil samples. The soil used in the experiment was leached chernozem with high humus content and low nitrate and ammonium nitrogen. Those properties indicated that the soil had been used for crop cultivation over a long period. The organomineral composition of soil determined the proportions of the main ecological-trophic groups of the soil microbial community and the dominant representatives of the microbial community. In the soil microbial community, the abundance of copiotrophs was 1.4 ± 0.5 million CFUs·g^−1^ soil, which was three times as low as the abundance of prototrophs and oligotrophs: 4.4 ± 0.9 and 4.3 ± 0.6 million CFUs·g^−1^ soil, respectively. The abundance of nitrogen-fixing microorganisms was 1.7 ± 0.2 million CFUs·g^−1^ soil. The high mineralization and oligotrophy coefficients along with the high abundance of nitrogen-fixing microorganisms were indicative of soil maturity and low contents of readily available nitrogen forms. Mineralization and oligotrophy coefficients reached 3.14 and 3.05, respectively. Analysis of the taxonomic composition showed that the bacterial community was dominated by Bacillus species, which constituted 65% of the isolates; they were followed by Actinobacteria—28% in total—and the Gram-negative Pseudomonas—7%. The total abundance of fungi was 14.7 ± 1.8 million CFUs·g^−1^ soil. The fungi were dominated by the typical representatives of soil microfungi—Penicillium (47.2%), Trichoderma (21.8%), and Mortierella (11.3%) species. Phytopathogenic species—potato pathogens Alternaria alternata (2.5%) and Fusarium oxysporum (7.8%)—were identified in soil samples.

**Figure 8:**
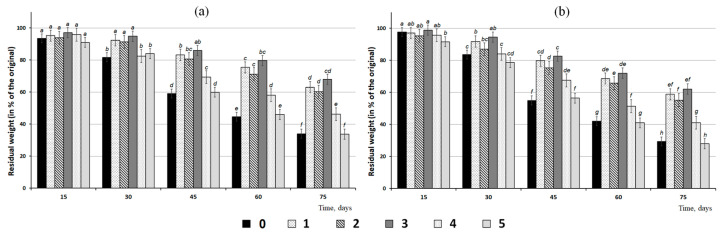
The weight loss of fungicide-loaded granules of two sizes ((**a**)—3.0; (**b**)—1.5 mm) incubated in soil: 0—unloaded formulation; 1—P(3HB)/wood flour/difenoconazole; 2—P(3HB)/wood flour/azoxystrobin; 3—P(3HB)/wood flour/prothioconazole; 4—P(3HB)/wood flour/azoxystrobin + mefenoxam; 5—P(3HB)/wood flour/mefenoxam. The letters indicate the significance of differences when comparing groups according to the Mann-Whitney test at the level of *p* < 0.05; identical letters indicate no significant differences.

A study was performed to investigate degradation behavior in soil of granules of two different sizes containing one of the four fungicides (difenoconazole, azoxystrobin, prothioconazole, or mefenoxam) or two fungicides (azoxystrobin + mefenoxam) (Figure 8). Fungicides were of different types and differed in their chemical composition and solubility. Solubility is a very important factor, as it obviously determines the release kinetics of the active ingredients. Mefenoxam had the highest solubility (up to 26 mg·L^−1^); azoxystrobin and difenoconazole had considerably poorer solubility (6.7 and 5 to 15 mg·L^−1^, respectively); and prothioconazole had the poorest solubility, which was largely determined by the pH of the medium: 0.005 g·L^−1^ at pH = 4 and much higher, 2.0 g·L^−1^, at pH = 9. 

P(3HB) degradation can be monitored not only by taking SEM images (Figure 5B) and recording the dynamics of the decrease in the total weight of the samples but also by measuring changes in the molecular weight parameters of the polymer. The initial weight average molecular weight and polydispersity of the polymer used to embed fungicides were 910 ± 20 kDa and 2.48 ± 0.3. The P(3HB) Mw and Đ decreased after the 75-d incubation in soil to 650 ± 60 kDa and 3.2 ± 0.5, respectively, suggesting the scission of carbon chains during polymer degradation.

As composite granules degraded in soil, their surface morphology was changing. Degradation caused the formation of pores, cracks, and voids in the amorphous regions of the polymer (Figure 5), and that resulted in a reduction in the total weight of the granules. Similar changes in the structure of the P(3HB-co-3HV)/wood flour composite were described in a study by Chan et al. (2019) [40]: as the composite was degraded by soil microorganisms, defects such as pores and cracks developed on the surface and in the bulk of the composite, which facilitated attachment and development of fungal microflora and, thus, increased degradation rate. The size of the granules and fungicide solubility influenced the weight loss dynamics of the fungicide formulations (Figure 8). 

During the first period (about 15 d), the granules were not degraded noticeably and no significant weight loss occurred. That delay was caused by the degradation behavior of polyhydroxyalkanoates, which undergo truly biological degradation—by microorganisms with extracellular PHA depolymerases [14,103,104]. The degradation phase of PHA-type polymers is preceded by a lag phase, which may last from a few days to one month or more, depending on environmental conditions. During the lag phase, microorganisms passivate the surface of the polymer samples and adapt to it; only after that, enzymes (PHA synthases) are synthesized, and the multistage polymer degradation process begins. That process involves gradual and sequential scission of carbon chains generating tetramers, dimers, and monomers of hydroxybutyric acid, which is consumed by microorganisms as growth substrate [105].

PHA biodegradation is one of the most valuable properties of these polymers, and, thus, it has been studied extensively. A great number of studies describe various species of microorganisms degrading PHAs; PHAs are known to be degraded in water, soil, sewage, seawater, mangrove forests, and activated sludge. Research shows that PHA degradation rate is determined by the polymer type (homopolymer P(3HB) or copolymers), the method of fabrication and geometry of polymer specimens, environmental conditions (various types of soil and water), the composition and activity of the microbial community, and climate and weather conditions. The half-life of PHA may vary between 30–60 d (in a tropical mangrove ecosystem [106], eutrophic reservoir [107], and Siberian [108] and tropical soil [109,110]) and 200–300 d or more (in compost [103], in tropical coastal waters [111], in a brackish lake [112], and in tropical soil [109,110]).

After 30 d of incubation of the granules with different fungicides in soil, their mass was 10–15% smaller than the initial mass. Degradation of unloaded granules, as well as those containing mefenoxam, was the most rapid (Figure 8). This trend was observed in subsequent stages of degradation. After 45 days, the mass of granules with mefenoxam and unloaded granules decreased by 40–45%, and after 60 days—by 55–60%, for large and small granules, respectively. On the same dates, the degradation of granules with other types of fungicides was significantly slower—the weight decreased by 20–35% of the original. At the end of the experiment (75 d), the residual mass of the larger granules was 33–46% of their initial mass at the minimum and 60–65% at the maximum. Degradation of the smaller granules was more noticeable: their residual mass varied between 30–40% and 55–58%. The granules containing the most readily soluble fungicide—mefenoxam—had the fastest degradation rate. The granules containing poorly soluble fungicides (difenoconazole, azoxystrobin, and prothioconazole) were degraded at a slower rate. The residual mass of these samples did not differ significantly and was notably higher than that of the samples with mefenoxam. At the same time, all fungicide-loaded granules were slow-release formulations with a half-life of 50 to 60 d, which, thus, were capable of sustained activity in soil and delivery of fungicides during the growing season. Similar degradation behavior was described for biocomposites of poly(3-hydroxybutyrate) and natural fillers (clay, peat, wood flour) in a study by Thomas et al. (2020) [62]. The authors of that study compared the degradation behavior of P(3HB)/natural material composites of two types: pellets produced by cold compaction from P(3HB) and filler material (wood flour, peat, or clay) powders and granules produced by spheronization of wet polymer/natural material paste. The study showed that degradation behavior was influenced by the geometry and technique of fabrication of the specimens; granules degraded in soil quicker than pellets. The literature data demonstrate that the degradation behavior of PHA/natural material composites is affected by the technique employed to process them. Specimens produced from melts are mechanically stronger and are degraded at a slower rate compared to the specimens produced by powder compaction. A team of researchers in Australia studied the properties of composites of several PHA types and wood flour of Australian pine [38,39,40]. Researchers [40] studied biodegradation of the P(3HB-co-3HV)/wood flour composites containing 0, 20, and 50 wt.% wood flour in soil in a subtropical region of Australia. The degradation rate of the composite increased with wood flour content, but the mechanical strength of the composite was not affected by wood flour content, decreasing by only 13% over 12 months. The degradation rate of the composite was five times greater than the degradation rate of the neat copolymer. Another study [113] also showed that composites of the degradable polymer P(3HB-co-3HV) and waste wood flour had high stability under controlled conditions in experiments with composite products incubated in soil 14 months—longer than neat copolymer products. Thus, PHA degradation in soil is a long process, which depends on various factors. 

Differences in the degradation behaviors of the fungicide formulations were reflected in the release dynamics of the fungicides and their concentrations in soil. Azoxystrobin and difenoconazole are relatively persistent pesticides, whose DT50 ranges between a few days and a few weeks or even months, as reported by different authors. The DT50 of mefenoxam is only a few days. Prothioconazole is also a quickly inactivated fungicide; its DT50 is 47.7 h under photodegradation in water, and the main product of prothioconazole photodegradation is prothioconazole-desthio (56%). Hence, fungicide concentrations in soil were determined not only by their solubility and release kinetics but also by their persistence in soil.

**Figure 9:**
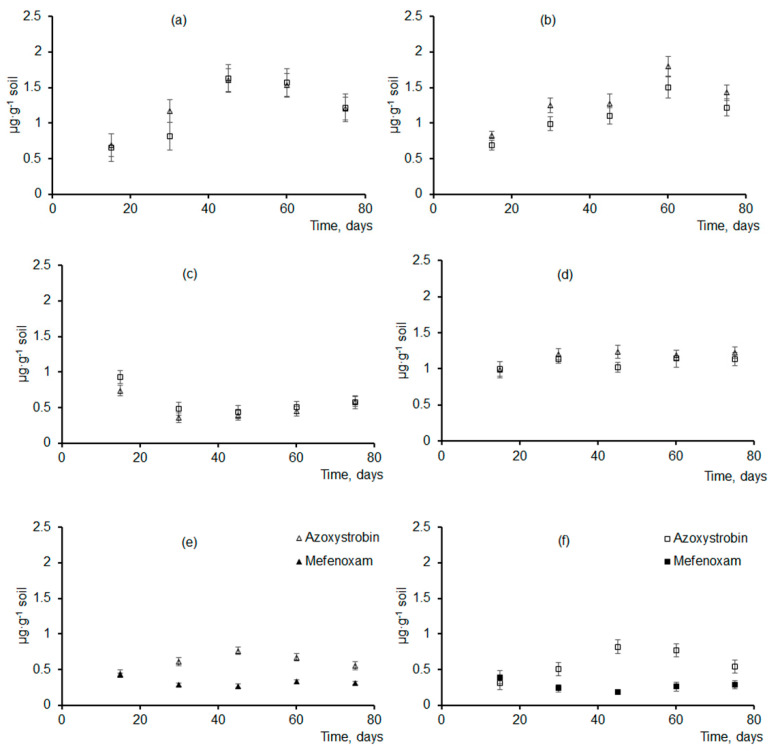
Current soil concentrations of fungicides (µg·g^−1^) released from experimental granules of different sizes: triangles—smaller granules (1.5 mm diameter); squares—larger granules (3.0 mm diameter). (**a**)—P(3HB)/wood flour/azoxystrobin, (**b**)—P(3HB)/wood flour/difenoconazole, (**c**)—P(3HB)/wood flour/mefenoxam, (**d**)—P(3HB)/wood flour/prothioconazole, (**e**)—P(3HB)/wood flour/azoxystrobin + mefenoxam (smaller granules), (**f**)—P(3HB)/wood flour/azoxystrobin + mefenoxam (larger granules).

The dynamics of fungicide accumulation in soil studied using two sizes of granules were comparable (Figure 9). First, fungicides were released from the granules due to passive dissolution and leaching from the surface of the active ingredients in the soil with the moisture content maintained at 50%. Then, fungicide release from the granules was mainly caused by the biodegradation of the polymer matrix. Azoxystrobin—a relatively stable but poorly water-soluble fungicide—was released gradually, and its concentration in soil 15 d after the granules were buried in soil (first measurement) was 0.6–0.7 µg·g^−1^ soil, reaching its maximum at days 45–60 and dropping after that.

Concentrations of other fungicides that were loaded into polymer matrix singly were comparable and changed in a similar fashion (Figure 9). Fifteen days after the beginning of the experiment, difenoconazole concentration was 0.7–0.8 µg·g^−1^ soil, increasing by a factor of 2 and reaching 1.5–1.8 µg·g^−1^ soil after 2 months. Prothioconazole and its main metabolite, prothioconazole-desthio, remained practically unchanged—1.0–1.3 µg·g^−1^ soil. The concentration of mefenoxam, the most readily water-soluble fungicide of the fungicides studied here, changed in a different way. Fifteen days after the beginning of the experiment (first measurement) mefenoxam concentration was 0.74 and 0.93 µg·g^−1^ soil (for the smaller and larger granules, respectively). By Day 30, however, mefenoxam concentration dropped to 0.36–0.48 µg·g^−1^ soil, probably because of the rapid degradation of the fungicide. That was in good agreement with the literature data on the DT50 of this fungicide: 5–6 d to 10–13 d, as reported by different authors. Then, mefenoxam concentration rose to its first values, consistent with the rapid degradation of the granules in that period. 

The release kinetics of the fungicides loaded together into the same granules (azoxystrobin + mefenoxam) were similar to their release kinetics from the granules containing each of them singly (Figure 9). The lower concentrations of these fungicides in soil were caused by their lower amounts in the initial granules. The total mass of both fungicides constituted 10% and 5% each. Note that the fungicide concentrations in soil were measured at definite time points of the experiment, and the amounts of the fungicides removed from soil or degraded in soil due to biogenic and abiogenic processes were not taken into account. However, soil is a complex and multifactor system, in which various biogenic and abiogenic processes influence the concentrations and “fate” of the chemicals applied to the soil. The chemicals are transformed by soil microflora in processes of co-metabolism and enzymatic oxidation. Furthermore, they are dissolved and degraded due to natural abiogenic processes occurring in the soil.

The release and accumulation of fungicides in soil affected the structure of the soil microbial community. Microbiological studies are important because microorganisms metabolize pesticides and serve as the major factor in transforming xenobiotics and removing them from the biosphere. An increase in pesticide concentrations leads to cumulative toxicity, resulting in suppression of microbial parameters of soil, reduction in diversity indices, and selection of resistant microorganisms [114,115,116,117]. In the present study, the comparison of the embedded and free fungicides applied to soil shows that, on the one hand, pesticides directly affect the structure of soil microbial community and metabolic potential of microorganisms and, on the other hand, soil application of fungicides embedded in P(3HB) matrix provides various soil bacteria and fungi with a supplementary growth substrate, stimulating their development.

During the experiment, as the granules were degraded and fungicide concentrations in soil were increased, the abundance of soil fungi was decreased (Figure S1). The abundances of plant pathogens of the genera *Fusarium* and *Alternaria* were below the detection limit from the middle to the end of the experiment, indicating effective suppression of plant pathogens in soil. The dominant fungi in all soil samples with the experimental fungicide formulations were representatives of the *Trichoderma* genus, whose proportion increased from 32 to 38%; *Mortierella* species increased by a factor of 2–2.6; by contrast, the percentage of *Penicillium* species decreased by a factor of 2 (Figure S2). The changes in the taxonomic composition of soil microflora were similar in the soil samples with granules of both sizes. 

As microbial activity determines the fertility of soil and its self-purification capacity, it was important to make sure that the experimental fungicide formulations neither exerted any negative effect on bacteria nor reduced their abundance and activity. The study of the effect of embedded fungicides on soil bacteria showed that during the first 1.0–1.5 months of the experiment, the abundance of bacteria in the soil with all experimental formulations was 10–20 times greater than in the negative control (Figure S3). That increase in abundance could be associated with both the effect of fungicides and reduced competition from the fungi, whose abundance was decreased. The greatest abundance of bacteria was observed in soil samples with mefenoxam granules (10–20 times greater than in the control) and difenoconazole (7.5–8.5 times greater). In soil samples with azoxystrobin granules, the abundance increased by a factor of 4–6, and in the soil with prothioconazole granules—by a factor of 2–2.5 compared to the control. Analysis of the taxonomic diversity of the bacterial community showed that the dominant bacteria in the control soil samples were *Bacillus* species (51%) and actinobacteria (36%). The microbial community of soil samples containing experimental fungicide formulations differed from that of the control soil because of the selective effect of fungicides on microflora (Figure S4). The general trend was a decrease in the proportion of spore-forming *Bacillus* species and an increase in the Gram-negative *Pseudomonas* and *Stenotrophomonas*. In addition, in soil samples with different fungicides, there were bacterial species that did not occur in other samples. For example, *Pseudomonas umsongensis* (19–23%), *Kocuria rosea* (9%), and *Streptomyces griseus* (4–5%) were identified in the soil with azoxystrobin granules; *Dermacoccus nishinomiyaensis* (8.5%) were isolated from the soil with mefenoxam granules; *Aeromonas schubertii* and *Clostridium cochlearium* were detected in the soil with prothioconazole and difenoconazole granules; and the dominant species in the soil with difenoconazole granules was *Bacillus sonorensis* (36%). Among the isolated bacteria, species previously described as P(3HB)-degraders were identified: *Bacillus cereus*, *Rhodococcus erythropolus*, *Stenotrophomonas maltophila*, and *Streptomyces* griseus [108,109,110]. P(3HB)-degraders of the genus *Pseudomonas* were also mentioned in the works by D. Jendrossek et al. [104,105]. The increase in the proportion of these microorganisms in the soil with experimental fungicide granules suggests the absence of an inhibitory effect of fungicidal preparations on P(3HB)-degrading bacteria and processes of decomposition of P(3HB) granules in soil.

Thus, incubation of the embedded fungicides in soil favored an increase in the abundance of bacteria at the low rates of release of the active ingredients and did not inhibit bacterial growth when the granules were degraded and fungicide concentrations in soil increased. However, the fungicides selectively affected the species composition of the soil bacterial community. At the same time, embedded fungicides effectively reduced the abundance of fungi in soil, exhibiting a strong antifungal effect on the plant pathogens *Alternaria* and *Fusarium*.

Results obtained in the present study are consistent with the literature data on the effect of the pesticide delivery method on the microbial community of field soil [118]. The authors of the study cited above reported that embedded pesticides had a considerably weaker negative effect on soil microflora, including functional groups of microorganisms and primary degraders of P(3HB), compared to free pesticides; moreover, sometimes, they produced a stimulating effect. Thus, prolonged-action pesticide formulations, which release pesticides gradually, preserve the degradation potential of microflora—the main factor preventing the accumulation of xenobiotics in the biosphere. The study of the effects of fungicides such as epoxiconazole, azoxystrobin, and tebuconazole demonstrated that the fungicides embedded in the P(3HB)/natural material (peat, clay, or wood flour) blends, like their free forms, controlled the growth of fungi and, hence, reduced the abundance of phytopathogenic fungi: *Alternaria*, *Pythium*, *Fusarium*, and *Verticillium*.

Analysis of the results obtained in the current study suggests that embedding of the study fungicides in the matrix consisting of P(3HB) blended with birch wood flour enables their gradual and slow release from the formulations, ensuring sustained delivery of fungicides to plants and pathogen suppression throughout the growing season. 

### 3.4. Biological Activity of Embedded Fungicides

The biological activity of fungicides was tested in vitro using five types of granules loaded with one of the fungicides (azoxystrobin, difenoconazole, mefenoxam, and prothioconazole) and two fungicides (azoxystrobin + mefenoxam). In the positive control, the active ingredients of these fungicides were used at the same concentrations. Experimental fungicide formulations fabricated as granules were tested in cultures of 10 strains of phytopathogenic fungi: *Alternaria alternata*, *Alternaria longipes*, *Boeremia exigua*, *Phytophthora infestans*, *Rhizoctonia solani*, *Fusarium oxysporum*, *Fusarium redolens*, *Fusarium solani*, *Fusarium equiseti*, and *Fusarium vanettenii*. These pathogens were isolated from the samples of the field soil and tubers of potato cv. *Krasnoyarskiy ranniy* and identified using conventional culture-based and molecular-genetic methods [81].

**Figure 10:**
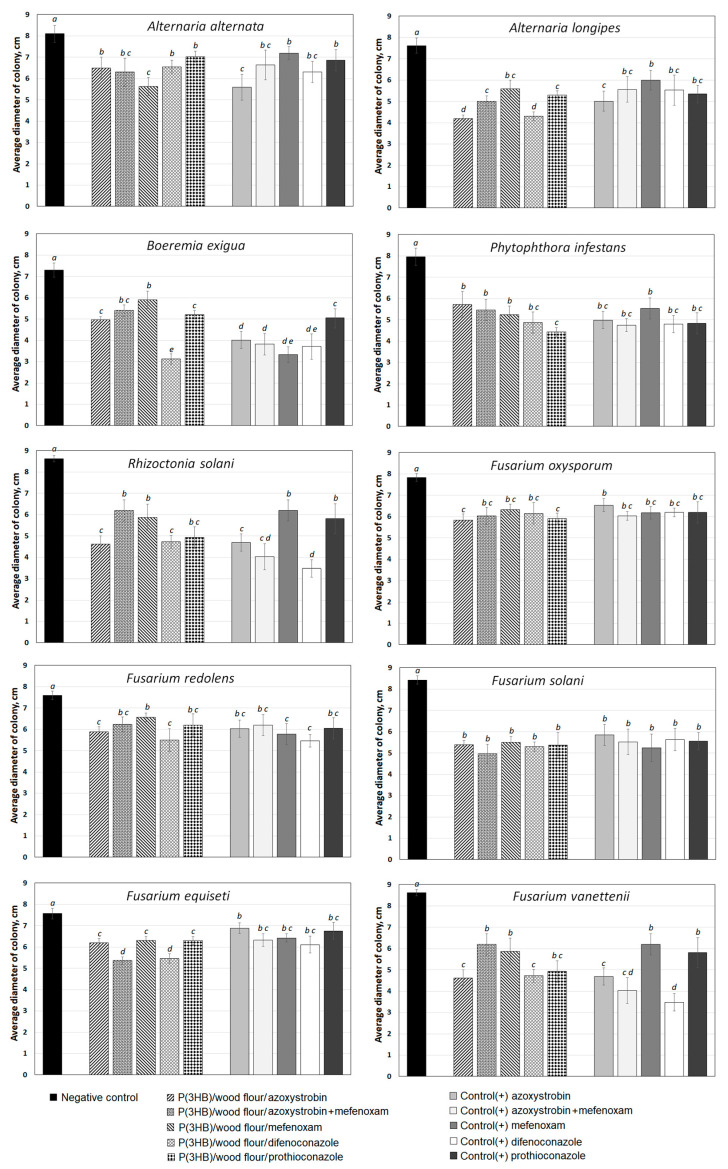
Diameters of the colonies of phytopathogenic fungi affected by different (embedded and free) forms of fungicides. The letters indicate the significance of differences when comparing groups according to the Mann–Whitney test at the level of *p* < 0.05; identical letters indicate no significant differences.

**Figure 11:**
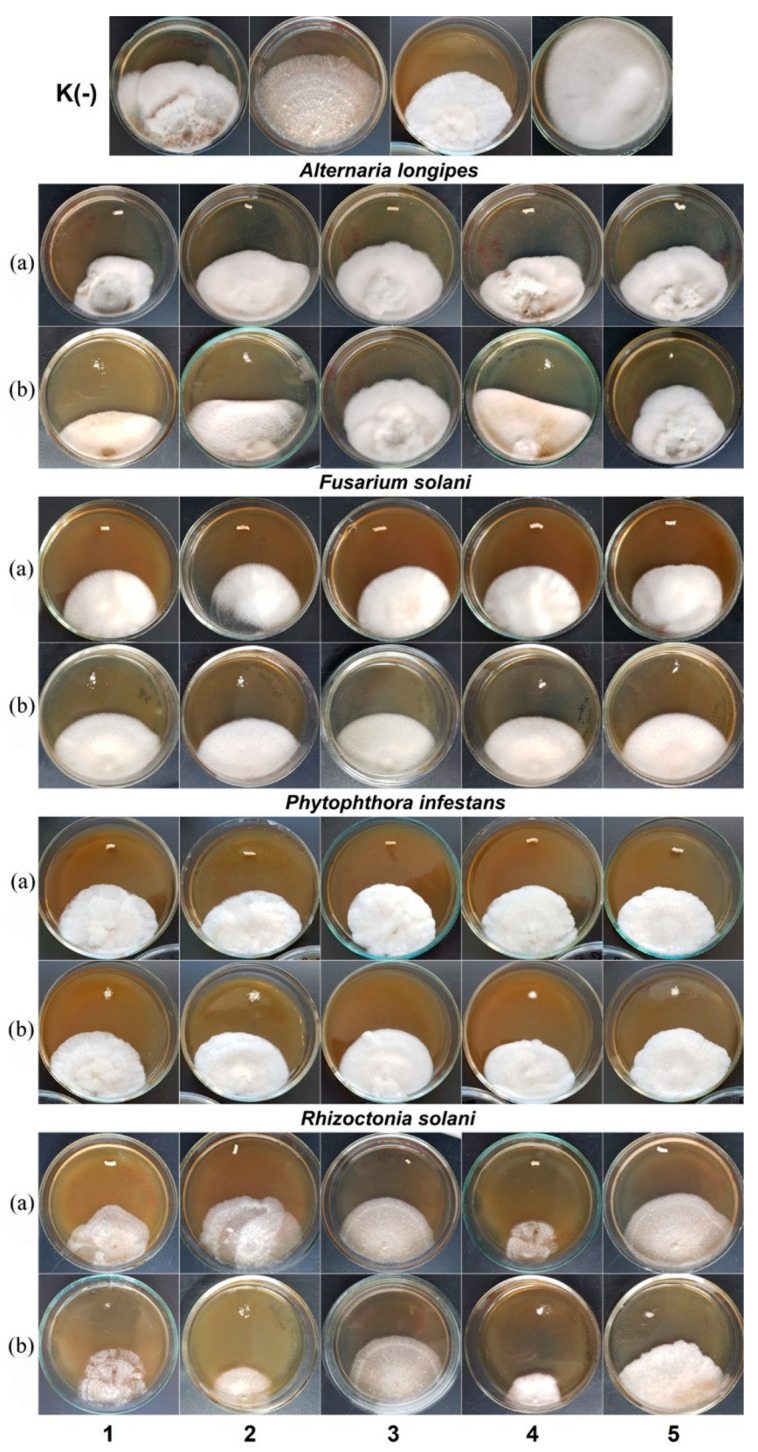
Growth of mycelium of phytopathogenic fungi on the fungicide-free medium (K(-)—negative control) and on the media with embedded (**a**) and free (**b**) fungicides: 1—azoxystrobin, 2—azoxystrobin + mefenoxam, 3—mefenoxam, 4—difenoconazole, 5—prothioconazole.

All experimental fungicide formulations produced an inhibitory effect on phytopathogenic microscopic fungi, which was comparable to the positive control (Figure 10 and Figure 11). Photographs of colonies of phytopathogenic fungi *A. longipes*, *F. solani*, *P. infestans*, and *R. solani* are given as examples in Figure 11. Similar results were observed for other species of phytopathogenic fungi. 

The embedded fungicides caused a 1.2- to 2.3-fold significant reduction in the average diameters of the colonies in different treatments relative to the negative controls, where there were no fungicides. The species that showed the highest sensitivity to the experimental fungicide formulations included *P. infestans*, *A. longipes*, *B. exigua*, *R. solani*, and *F. solani*. Among the embedded fungicides, granules with difenoconazole had the maximum inhibitory effect, azoxystrobin was in second place, and the complex formulation azoxystrobin + mefenoxam was in third place.

In comparison with the positive control, free forms of fungicides (the active ingredient of the fungicide in the amount equivalent to its amount in the granule) showed that in most cases, the effectiveness of the embedded fungicides was comparable to that of the free fungicides, with the exception of the species *B. exigua*, *R. solani*, and *F. vanettenii*, against which free forms of fungicides containing difenoconazole or azoxystrobin + mefenoxam showed a stronger fungicidal effect (Figure 10). At the same time, the effectiveness of the embedded fungicide was chiefly determined by the level of sensitivity of the fungus, probably due to species characteristics. For instance, of the *Alternaria* species, *A. alternata* was affected by both free and embedded fungicides to a lesser extent than *A. longipes*. A similar trend was observed for Fusarium: F. redolens and F. oxysporum species were less sensitive to all fungicides than *F. solani* or *F. vanettenii* species.

The average diameter of the intact *A. longipes* colonies in the negative control (without fungicides) reached 7.6 ± 0.4 cm. In the presence of the embedded azoxystrobin and difenoconazole, the diameter of the colony was reduced by a factor of 1.8; in the presence of mefenoxam and prothioconazole—by a factor of 1.4. The free fungicides controlled fungal growth equally effectively, reducing the diameter of the colony by a factor of 1.4–1.6. In most cases, inhibition of *A. alternata* colonies growth by embedded and free forms did not differ significantly.

In the negative control, the *F. solani* colony covered almost the entire surface of the agar medium in the Petri dish, and its diameter was 8.8 ± 0.2 cm (Figure 11). All experimental fungicide formulations were equally effective in inhibiting the growth of this pathogen: the diameters of the colonies affected by fungicides were decreased by a factor of 1.7–1.8. Similar results were obtained for the phytopathogenic fungus *P. infestans*. In the negative control, with no fungicides, the diameter of the colony was 7.1 ± 0.4 cm. Experimental fungicide formulations reduced the diameter of the colonies by a factor of 1.4–1.8. The effects of the free and embedded fungicides did not differ significantly. The phytopathogenic fungus *R. solani* was the most sensitive to the action of fungicides. Through exposure to free difenoconazole, the diameter of the colonies was reduced relative to the control by a factor of 2.3. The experimental granules containing azoxystrobin also had a significant inhibitory effect on *R. solani* mycelial growth. The effects of the embedded and free prothioconazole and mefenoxam did not differ significantly, reducing the diameter of the colony by a factor of 1.4 relative to the negative control. 

The formulation of the azoxystrobin fungicide embedded in the matrix of P(3HB) blended with natural fillers (wood flour, peat, or clay) was fabricated and studied previously [65]. The biological activity of the embedded azoxystrobin was tested and confirmed in vitro, in the culture of the phytopathogenic fungus Fusarium verticillioides, causing root rots of cereal crops. All other embedded fungicides were tested in this study in vitro, in the cultures of plant pathogens causing potato diseases, for the first time. All experimental formulations had a fungicidal effect and showed high biological activity against the most harmful potato pathogens (*P. infestans*, *R. solani*, and *F. solani*), which was comparable to the activity of free fungicides.

## 4. Conclusions

Slow-release fungicide formulations for suppressing potato pathogens were constructed using a homogenized blend of the biodegradable poly(3-hydroxybutyrate) [P(3HB)] powder and natural filler—birch wood powder. Systemic contact fungicides with different modes of action (difenoconazole, mefenoxam, prothioconazole, and azoxystrobin) were studied. The slow-release fungicide formulations were fabricated as granules of two sizes (1.5 and 3.0 mm in diameter) by spheronization technique, using a polymer/wood flour/fungicide mixture (50/40/10 wt.%). All initial materials (polymer, wood flour, and fungicides) and fungicide-loaded granules were analyzed using DTA, DSC, X-ray analysis, and SEM. To reveal the type of interactions between the components in the granules, the IR spectra of the granules were compared to the IR spectra of all initial materials. Analysis of IR spectra showed that no chemical bonds were established between the polymer, wood flour, and fungicides and that the composites produced were physical mixtures. Filling of P(3HB) with wood flour and fungicides reduced the degree of crystallinity and changed the thermal properties of the polymer. The thermal properties and degrees of crystallinity of the experimental fungicide formulations were lower than those of P(3HB). Biodegradation of composite granules in soil was studied for 75 days in laboratory micro-ecosystems with the pre-characterized field soil at a stable temperature and moisture content. The study showed that the experimental slow-release fungicide formulations were prolonged-action forms with a half-life of at least 50–60 d, enabling gradual and sustained delivery of the active ingredients to plants. The size of the granules and the structure of the fungicides, which differed in their solubility, affected the degradation behavior of the granules. The biological activity of fungicide formulations was studied in the culture of pathogenic fungi using the disk diffusion method, and results showed that all experimental formulations had a strong inhibitory effect on the most common and harmful potato pathogens (*Phytophthora infestans*, *Alternaria longipes*, *Rhizoctonia solani*, and *Fusarium solani*). Thus, the present study demonstrated for the first time that embedding of the fungicides in the matrix consisting of poly(3-hydroxybutyrate) blended with birch wood flour enables their gradual and slow release from the formulations, ensuring sustained delivery of fungicides to plants and pathogen suppression throughout the growing season.

## Figures and Tables

**Table 1:** Characterization of the fungicides studied [78].

Name of Fungicide	Structural Formula	Use
Difenoconazole (a triazole fungicide); purity ≥ 95.0%	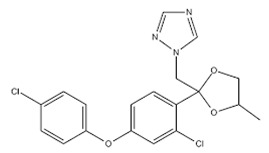	Effective against ascomycetes, basidiomycetes, deuteromycetes, common bunt, and root rots; protects the potato from black scurf, silver scab, fusarium disease, black dot
Mefenoxam (a phenylamide fungicide); purity ≥ 91.0%	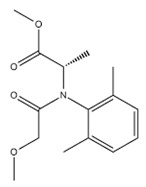	Used to control potato diseases (late blight, early blight); used alone and mixed with other active ingredients to control plant diseases
Prothioconazole (a triazole fungicide); purity ≥ 98.0%	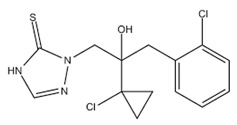	Effective against a wide range of plant pathogens (ascomycetes, basidiomycetes, deuteromycetes, common bunt, and root rots); protects the potato from black scurf, silver scab, fusarium disease
Azoxystrobin (a strobilurin fungicide); purity ≥ 98.0%	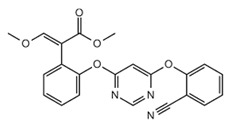	Suppresses a broad variety of pathogens. Protects the potato from black scurf, early blight, late blight

Difenoconazole [cis-trans-3-Chloro-4[4-methyl-2-(1H-1,2,4-triazol-1-ylmethyl)-1,3-dioxolan-2-yl]phenyl 4-chlorophenyl ether]; Mefenoxam [(R)-enantiomer of metalaxyl] [Methyl-N-(methoxyacetyl)-N-(2,6-xylyl)-D-alaninate]; Prothioconazole [2-[(RS)-2-hydroxy-2-(1-chlorocyclopropyl)-3-(2-chlorophenyl)propyl]-2H-1,2,4-triazol-3(4H)-thione]; Azoxystrobin [Methyl(E)-2-{2-[6-(2-cyanophenoxy)pyrimidin-4-yloxy]phenyl}-3-methoxyacrylate].

**Table 2:** Physico-chemical properties of the initial fungicides, materials of the matrix, and experimental fungicide formulations.

Sample	C_x_, %	T_melt_, °C	T_degr,_ °C	Enthalpy of Melting, (J·g^−1^)
Fungicides				
Difenoconazole	56	78.6	329	60.2
Azoxystrobin	66	118	315	86.2
Mefenoxam	-	140	288	37.0
Prothioconazole	63	-	236	-
Materials used to construct matrix for embedding fungicides				
P(3HB) polymer	76	170	282	89.1
Birch wood flour	26	-	220 *	-
Composition and properties of experimental formulations				
P(3HB)/wood flour/difenoconazole	43	78 139	275 343	5.6 51.7
P(3HB)/wood flour/azoxystrobin	55	118 164	274 347	7.4 44.1
P(3HB)/wood flour/mefenoxam	43	141 168	275 360	3.6 43.9
P(3HB)/wood flour/azoxystrobin + mefenoxam	56	117 137 171	275 348	2.7 1.3 29.2
P(3HB)/wood flour/prothioconazole	42	173	277	46.4
P(3HB)/wood flour	58	174	276 348	57.5

“-” denotes that the sample does not have this parameter; *—onset of thermal decomposition.

## Data Availability

All data are available in the paper.

## Supplementary Materials

The Supplementary Materials are available online at https://www.mdpi.com/article/10.3390/polym14173669/s1.
