# Supplementary figures and images for: Degradable Poly(3-hydroxybutyrate)—The Basis of Slow-Release Fungicide Formulations for Suppressing Potato Pathogens
Source: Polymers (Basel). 2022 Sep 3;14(17):3669. doi: 10.3390/polym14173669 (PMC9460056; doi:10.3390/polym14173669)

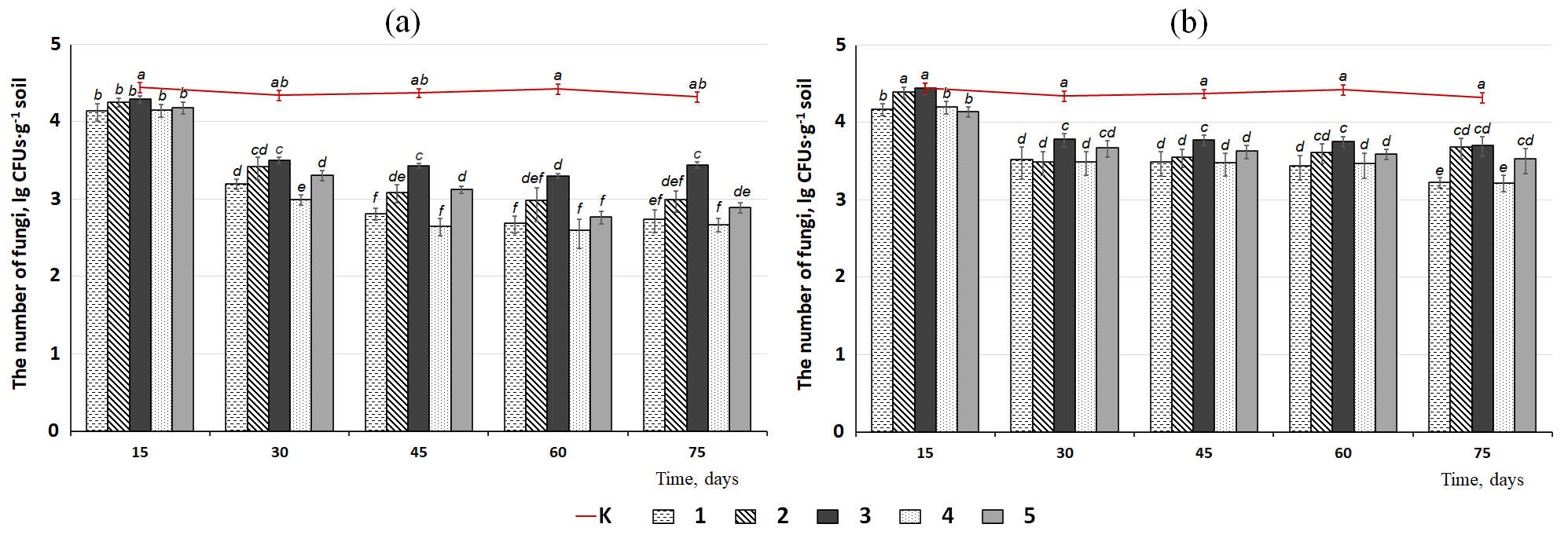

Supplement: Supplementary file 1 [file polymers-14-03669-s001.zip › Figure S1.tif]

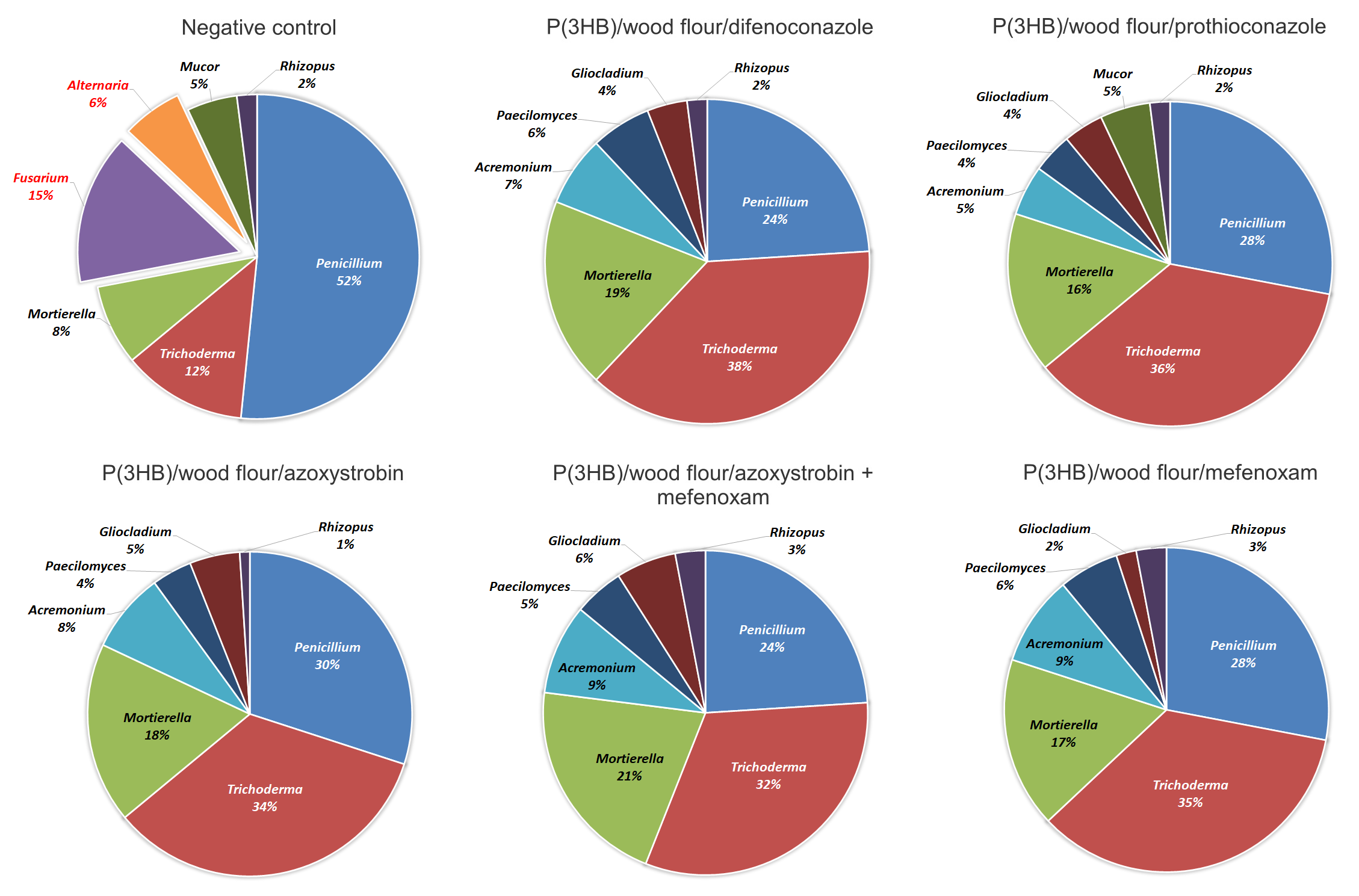

Supplement: Supplementary file 1 [file polymers-14-03669-s001.zip › Figure S2.tif]

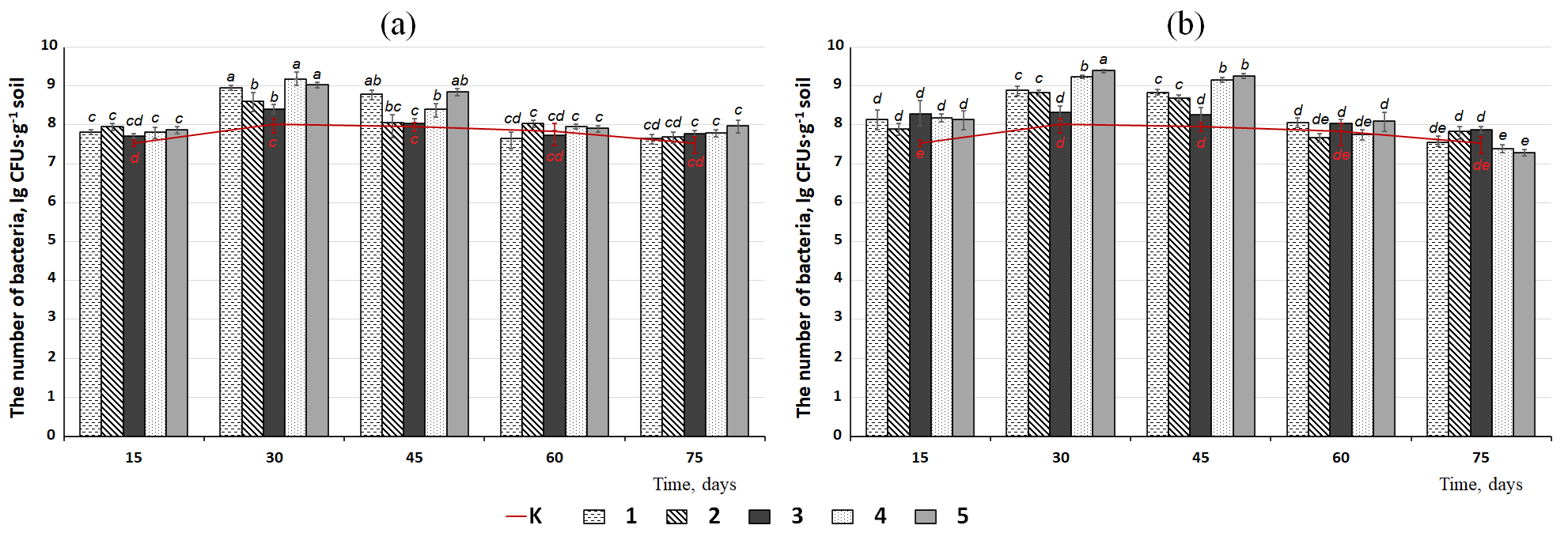

Supplement: Supplementary file 1 [file polymers-14-03669-s001.zip › Figure S3.tif]

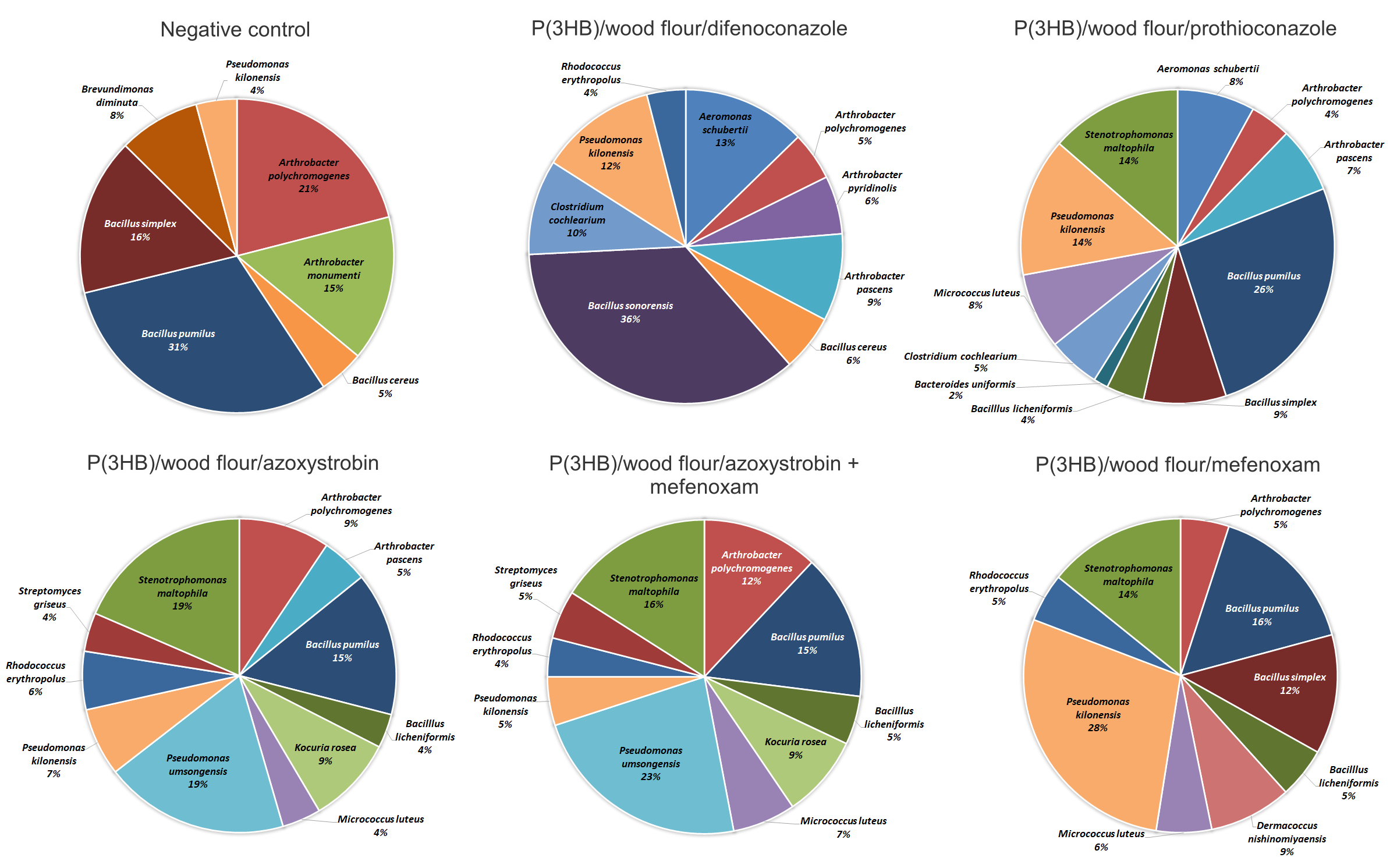

Supplement: Supplementary file 1 [file polymers-14-03669-s001.zip › Figure S4.tif]
